# A comprehensive sensitivity analysis of microarray breast cancer classification under feature variability

**DOI:** 10.1186/1471-2105-10-389

**Published:** 2009-11-26

**Authors:** Herman MJ Sontrop, Perry D Moerland, René van den Ham, Marcel JT Reinders, Wim FJ Verhaegh

**Affiliations:** 1 Molecular Diagnostics Department, Philips Research, High Tech Campus 12a, 5656 AE Eindhoven, the Netherlands; 2 Bioinformatics Laboratory, Department of Clinical Epidemiology, Biostatistics and Bioinformatics, Academic Medical Center, Meibergdreef 9, 1100 AZ Amsterdam, the Netherlands; 3 Biomolecular Engineering Department, Philips Research, High Tech Campus 11, 5656 AE Eindhoven, the Netherlands; 4 Delft Bioinformatics Lab, Delft University of Technology, Mekelweg 4, 2628 CD Delft, the Netherlands

## Abstract

**Background:**

Large discrepancies in signature composition and outcome concordance have been observed between different microarray breast cancer expression profiling studies. This is often ascribed to differences in array platform as well as biological variability. We conjecture that other reasons for the observed discrepancies are the measurement error associated with each feature and the choice of preprocessing method. Microarray data are known to be subject to technical variation and the confidence intervals around individual point estimates of expression levels can be wide. Furthermore, the estimated expression values also vary depending on the selected preprocessing scheme. In microarray breast cancer classification studies, however, these two forms of feature variability are almost always ignored and hence their exact role is unclear.

**Results:**

We have performed a comprehensive sensitivity analysis of microarray breast cancer classification under the two types of feature variability mentioned above. We used data from six state of the art preprocessing methods, using a compendium consisting of eight diferent datasets, involving 1131 hybridizations, containing data from both one and two-color array technology. For a wide range of classifiers, we performed a joint study on performance, concordance and stability. In the stability analysis we explicitly tested classifiers for their noise tolerance by using perturbed expression profiles that are based on uncertainty information directly related to the preprocessing methods. Our results indicate that signature composition is strongly influenced by feature variability, even if the array platform and the stratification of patient samples are identical. In addition, we show that there is often a high level of discordance between individual class assignments for signatures constructed on data coming from different preprocessing schemes, even if the actual signature composition is identical.

**Conclusion:**

Feature variability can have a strong impact on breast cancer signature composition, as well as the classification of individual patient samples. We therefore strongly recommend that feature variability is considered in analyzing data from microarray breast cancer expression profiling experiments.

## Background

Microarrays are a powerful tool for biologists as they enable the simultaneous measurement of the expression levels of thousands of genes per tissue sample [1]. One of the interesting applications of gene expression profiling is the identification of compact gene signatures for diagnostic or prognostic purposes, such as cancer classification. One of the first studies in this regard was the work of Van 't Veer et al. [2], in which a prognostic 70-gene signature is identified, that can be used to assess whether a breast tumor is likely to metastasize or not. Signatures like the 70-gene signature of Van 't Veer are, in essence, comprised of two parts: a limited set of features and a classifier that maps a vector of feature values to a class label. Limiting the number of features has several advantages. For one, using too many features with flexible classifiers quickly leads to overfitted decision rules. The inclusion of irrelevant features can also substantially degrade the performance of some classifiers. Furthermore, understandability, efficiency, and cost also benefit from more compact rules.

Microarray breast cancer event prediction, however, has proven to be difficult, as few classification rules are able to obtain a balanced accuracy rate of over 70%, when properly validated [3,4]. These performance indicators are also often associated with wide confidence intervals [5]. Furthermore, Ein-Dor et al. [6] showed that signature composition strongly depends on the subset of patient samples used for feature selection. In recent years many different signatures have been proposed, mostly derived using different patient populations and/or array technologies. Although the overall performance of these signatures is comparable, there is often a high level of inconsistency between class assignments obtained using different signatures, as was recently reported in [7]. This poses significant challenges for the use of gene expression classifiers in clinical routine. Although biological variability is conjectured to play a major role in the observed discrepancies, in this paper we show that even in a very controlled setting, using identical arrays, patient samples, signature composition, and classifiers, still large discrepancies in performance and individual class assignments can be observed under two types of variability.

One of the challenging aspects of microarray data is that they are subject to various sources of technical variation, arising from the many experimental laboratory steps needed to get from a tissue sample to an array scan, such as array batch variability, dye incorporation, uneven hybridizations, probe-failure caused by dust or scratches, or washing conditions [8]. Some noise factors bias large groups of measurements in a systematic way. Fortunately, most of this bias can be removed by proper preprocessing. Many preprocessing methods have been proposed to address these systematic biases. The effectiveness of such procedures and the plausibility of their assumptions, however, depends on factors such as study design, the array technology being used, and the biological phenomenon under study [9]. Furthermore, even after correction for systematic effects by the preprocessing method, there remains a residual variance that is both array and feature specific and that can be substantial [10]. Detailed error models have been proposed that attempt to quantify such uncertainty around the expression data point estimates, e.g. the Rosetta error model [11]. Such uncertainty information has been incorporated in differential gene expression analysis methods [12], as well as in clustering analysis [13], and principal component analysis [14], often leading to more consistent results.

The impact of noise on the outcome of the statistical analysis of microarray data has been a subject of debate. Tu et al. [15] performed a detailed sensitivity analysis to separate noise caused by sample preparation from noise related to the hybridization process. The latter was identified to be the more dominant of the two. In addition, a strong dependence of hybridization noise on the expression level was reported. Based on data from the MAQC study [16], however, Klebanov et al. [17] claim that for Affymetrix arrays the magnitude of technical variation has been gravely exaggerated in the literature and that the effects on the results of statistical inference from Affymetrix GeneChip microarray data are negligibly small. However, contradictory findings have been reported in [18], based on the very same data. In addition, the MAQC study itself has been criticized for presenting their case in a best case scenario, using too few and overly clean reference samples [19]. With regard to the impact of the choice of preprocessing method, it has been observed in differential expression studies that preprocessing can strongly influence whether a gene is detected to be differentially expressed or not [20,21]. Similar observations have been made for the influence of preprocessing on classification [22,23], albeit in a different and much smaller setting than the work presented here.

Although microarray data is known to be subject to the sources of variation described above, in microarray breast cancer classification studies the influence of the choice of preprocessing scheme and of the uncertainty around expression data point estimates are almost always ignored. In this paper, we study the effect of these two types of variability of expression data on breast cancer classification in detail. We define *preprocessing variability *as the variation in the value of a feature as induced by switching to an alternative preprocessing scheme. *Perturbation variability *is defined as the variation in the value of a feature as caused by adding noise based on the uncertainty information associated with the expression data point estimates. Furthermore, *feature variability *is understood to be the variation in the value of a feature as caused by either preprocessing or perturbation variability.

We have performed a comprehensive sensitivity analysis of microarray breast cancer classification under feature variability. We used a large breast cancer compendium consisting of eight different datasets, involving 1131 hybridizations, and containing data from both one and two-color array technology. We studied the impact of preprocessing and perturbation variability on feature selection, classification performance, and classification concordance for six different preprocessing methods. In addition, we performed a comprehensive stability analysis for a diverse set of classifiers, by explicitly testing these classifiers for their noise tolerance. Stability was quantified by the variation in class assignment of perturbed expression profiles, where the amount of perturbation is based on uncertainty information directly related to the selected preprocessing strategy. Our results indicate that even when using identical arrays and sample populations, preprocessing and perturbation variability have a strong impact on the classification of individual breast cancer samples, as well as on the composition of breast cancer signatures, especially when the number of features is low.

## Methods

### Data

The datasets we consider in this paper share a common theme, i.e., they have been used to predict whether a breast tumor will metastasize within five years (poor prognosis) or not (good prognosis), based on gene expression data inferred from removed tumor tissue. We performed our sensitivity analysis using a compendium of eight publicly available datasets. In total, the compendium contains microarray data from 1131 hybridizations and for 907 samples class label information was available (Table 1). Some of the eight datasets initially had an overlap, either in patient samples or in hybridizations. The compendium of 907 arrays, however, contains no overlap, as all duplicate cases were removed. Data from the studies of Van 't Veer and Van de Vijver were obtained using two-color custom ink-jet oligonucleotide arrays produced by Agilent. Processed data for these datasets can be downloaded from http://www.rii.com/publications/2002/default.html. Like the original authors, we combined the two datasets. We refer to this combined dataset as the Rosetta dataset. The Rosetta dataset consists of 87 lymph-node negative samples of the Van de Vijver dataset and of the 78 training and 19 validation samples of the Van 't Veer dataset. Data for all other datasets was obtained using Affymetrix GeneChips and CEL files were downloaded from GEO [24] and ArrayExpress [25]. A more comprehensive overview of the selected hybridizations for the Affymetrix datasets, including class label information, can be found in Additional File 1. Additional File 2 gives an overview of the 87 cases of the Van de Vijver dataset that were added to the Van 't Veer dataset.

**Table 1:** Dataset overview

*author*	*year*	*total*	*labeled*	*Good*	*poor*	*array platform*	*repository*	*accession*	*ref*
Desmedt	2007	147	120	91	21	Affymetrix HG-U133A	GEO	GSE 7390	[49]
Minn	2005	96	62	41	21	Affymetrix HG-U133A	GEO	GSE 2603	[50]
Miller	2005	247	193	156	37	Affymetrix HG-U133A	GEO	GSE 3494	[51]
Pawitan	2005	156	142	120	22	Affymetrix HG-U133A	GEO	GSE 1456	[52]
Loi	2007	178	120	92	28	Affymetrix HG-U133A	GEO	GSE 6532	[53]
Chin	2006	123	86	63	23	Affymetrix HG-U133A	ArrayExpress	E-TABM-158	[54]
Van't Veer	2002	97	97	51	46	Agilent 2-color custom	-	-	[2]
Van de Vijver	2002	87	87	75	12	Agilent 2-color custom	-	-	[34]

The column *total *contains the total number of hybridizations available, while the column *labeled *shows the number of samples that have a properly defined class label. The next two columns indicate the decomposition of this number into good and poor prognosis cases. The columns *repository *and *accession *list in what repository and under which accession number each dataset can be found.

### Preprocessing

For the Van 't Veer and Van de Vijver datasets, we used the publicly available expression estimates and corresponding error information based on the Rosetta error model [11]. In principle, the Rosetta error model is applicable to both one and two-color arrays. However, for this model no freely available implementation exists and hence for the Affymetrix datasets this model was not applied. For the datasets using Affymetrix GeneChips we generated expression data from the available CEL files based on five different, frequently used preprocessing strategies: MAS 5.0, mgMOS, its multi-chip version mmgMOS, RMA, and dChip. For preprocessing, all available hybridizations were used. This is especially relevant for the multi-chip models dChip, RMA, and mmgMOS, which benefit from having more arrays assuming all hybridizations are of similar quality. The dChip expression estimates are constructed using only the information of the PM-probes, which is the default choice for dChip. Affymetrix datasets were log-transformed and all probesets were median centered after preprocessing, for each dataset separately. The validity and benefits of this step are further discussed in [4] and [26]. Preprocessing for the Affymetrix datasets was performed in R [27] using Bioconductor [28] packages affy [29] and puma [30]. Table 2 provides a summary of the six preprocessing methods used.

**Table 2:** Preprocessing overview

*method*	*package*	*function*	**log**_**2**_	*σ*	*reference*
RMA	affy	Expresso	yes	yes	[55]
mgMOS	puma	justmgMOS	yes	yes	[56]
mmgMOS	puma	justmmgMOS	yes	yes	[57]
dChip	affy	expresso	no	yes	[58]
MAS5.0	affy	expresso	no	no	[59]
Rosetta	-	-	-	-	[11]

The column *package *indicates which R package was used to obtain the expression values, while the column *function *provides the name of the function used from the package. Column log_2 _indicates if the expression estimates as returned by the function are already on log_2 _scale or not. The column *σ *indicates if the function directly computes uncertainty information or not.

### Perturbation

After preprocessing, we get an expression estimate *x*_*ij *_for each array *i *and each feature (gene) *j*. In fact, *x*_*ij *_is usually stochastic, following some distribution *D*_*ij *_with mean *μ*_*ij *_= *x*_*ij *_and standard deviation *σ*_*ij *_reflecting the measurement uncertainty associated with the point estimate *x*_*ij*_. In this paper, we utilized the uncertainty information as captured by the distributions *D*_*ij *_to generate perturbed expression profiles as alternatives for expression point estimates *x*_*ij*_, in a similar fashion as presented in [13]. For each sample *i*, for each gene *j *in a given signature, we simply draw a new data point  by sampling from the corresponding distribution *D*_*ij*_. Complete perturbed training and validation sets can be constructed by repeating this process for all samples and genes.

The Rosetta model, mgMOS, and mmgMOS are specifically designed to provide a *σ*_*ij *_that reflects the uncertainty of the complete preprocessing cascade. In these three models *D*_*ij *_is a Gaussian distribution. For mgMOS and mmgMOS, the corresponding *σ*_*ij *_values were obtained using the R package puma[30]. For the Van 't Veer and Van de Vijver datasets, we used the published expression values. For the Van de Vijver data, the standard deviations *σ*_*ij*_, as estimated by the Rosetta error model, were reported directly. For the Van 't Veer data *σ*_*ij *_was not provided directly, but *σ*_*ij *_could be recovered from the published *p*-value information (see Additional File 3). MAS 5.0, dChip and RMA are not specifically designed to provide detailed error estimates, although some of the uncertainty associated with the point estimates can be derived from the summarization step in the preprocessing cascade. For RMA and dChip, the uncertainty corresponding to the summarization step can again be modeled by a Gaussian distribution. The estimated *σ*_*ij *_values for these two models were obtained using the R package affy[29]. For MAS 5.0 it turns out that the estimates follow a distribution closely related to a *t*-distribution. Although error information for MAS 5.0 is not available from affy directly, it can be computed from the information affy provides (see Additional File 3).

### Stability measure: minority assignment percentage

Classification instability occurs when for a given classifier and a given sample, perturbed expression profiles are not all assigned to the same class. In order to quantify the instability over a large number of perturbed datasets, we propose the following simple stability measure, which we refer to as the *minority assignment percentage (map) *score. For a given sample and feature, we denote the percentage of perturbed datasets that lead to a classification into class 0 by *p*_0_, and the percentage leading to a class label 1 by *p*_1_. Then the minority assignment percentage is equal to min{*p*_0_, *p*_1_}. In the ideal case, a map-score is equal to zero, indicating that all perturbed datasets lead to the same classification for this specific sample. In the worst case, it equals 50%, indicating that classification is purely random. Note that this observation is independent of the choice of dataset, perturbation mechanism, classifier or number of features. In the remainder of the paper we will consider a classification to be unstable if the map-score exceeds a conservative threshold of 35%, meaning an almost random classification.

### Sensitivity analysis protocol

**Figure 1:**
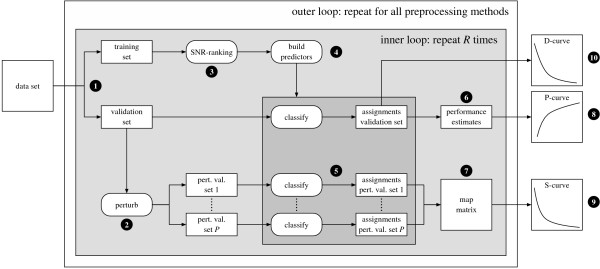
**Sensitivity analysis protocol**. For an explanation, see the running text.

All classification results are obtained in a systematic fashion, closely related to the protocol proposed in [3]. Figure 1 provides a schematic overview of our workflow. Assume we have obtained expression values *x*_*ij *_and the corresponding *σ*_*ij *_values, for a given measure of expression, for some set of samples and a set of genes, using the methods described in the previous sections. In addition, assume we have selected an appropriate classifier, which we need to train. In the first step, we create a stratified split of the available data, in which 80% is used as a training set, while the remaining 20% serves as a validation set. In step 2, we create *P *= 1000 perturbed versions of the validation set. In step 3, we rank the features based on their Signal-to-Noise Ratio (SNR, see next section) on the (unperturbed) training set. In the next step, we use the top-100 ranked features to construct a sequence of 100 classifiers, where the *n*^th ^classifier is constructed on the training data, using only the top-*n *ranked genes. At step 5, we invoke each classifier to obtain class assignments for both the unperturbed validation set, and for all perturbed versions. In step 6, we obtain a performance estimate for the unperturbed validation data by computing the balanced accuracy rate, that is, the average of the sensitivity and specificity. In step 7, we use the class assignments of the 1000 perturbed validation sets, as obtained in step 5, to compute the associated map-scores and collect them in a *map-matrix*, where the entry at row *i *and column *n *represents the map-score of validation sample *i*, for a classifier trained on the top-*n *ranked features. To ensure that results are not split-specific, steps 1 to 7 are repeated *R *= 50 times (inner loop). At step 8, we compute a performance curve, referred to as the *P-curve*, which for each signature size *n *∈ {1,...,100}, displays the average balanced accuracy over the *R *splits. Furthermore, as mentioned, for a given sample we consider a class assignment to be unstable if the corresponding map-score is larger than some threshold *T *= 35. For a given threshold, in step 9 we compute the stability curve, referred to as the *S-curve*, which for each signature size tells us the average percentage of cases, over *R *splits, that had a map-score larger than the selected threshold *T*. Note that ideally the S-curve should be zero for all entries. The whole procedure described above is repeated for each preprocessing method (outer loop). In order to compare results for different classifiers and preprocessing methods, for a given dataset and for each repeat of the inner loop we always used the same set of stratified splits. Finally, in step 10 we generate a discordance curve, referred to as the *D-curve*, for all distinct preprocessing method pairs. For a preprocessing method pair (*m, m'*) and given classifier, the corresponding D-curve tells us for each signature size the average percentage of cases, over *R *splits, of inconsistent class assignments on the (unperturbed) validation sets. Similarly to the S-curve, ideally a D-curve is zero for all entries. Note that the map-scores used for the S-curves can also be viewed as a measure of concordance, under perturbation variability.

### SNR-based feature rankings

As stated in previous section, in the third step of our protocol we rank the available features based on their signal-to-noise ratios. For a given feature, let *μ*_0 _and *μ*_1 _denote the mean intensity value for class 0 and class 1, respectively, and let *σ*_0 _and *σ*_1 _be the corresponding standard deviations. Then the SNR is equal to(1)

Let SNR_*j*, *m *_denote the SNR value corresponding to gene *j*, based on data corresponding to preprocessing method *m*. In the construction of a signature we typically select the top-*n *features from such a ranking.

Let *F*_*n*, *m *_denote the top-*n *genes, obtained using data from preprocessing method *m*, for a particular split. Different preprocessing methods may lead to different lists of top-*n *genes. For two different methods *m *and *m'*, a trivial measure to compare the lists *F*_*n*, *m *_and *F*_*n*, *m' *_would be to look at their intersection. From a classification standpoint, however, we would at least hope to obtain two lists that are of comparable strength. Let the total strength of a feature set *F *with respect to method *m *be defined as(2)

To compare two gene lists of cardinality *n*, we introduce the concept of *relative strength*, given by(3)

The relative strength compares the total strength with respect to *m *for a selection based on *m' *to the selection based on preprocessing method *m *itself. As the latter gives the maximal total strength for a set of size *n *with respect to method *m*, the resulting relative strength will always be at most 100.

Furthermore, since SNR values are non-negative, the relative strength is also non-negative. Note that a high relative strength implies that we expect a similar performance when using *F*_*n*, *m' *_as when using *F*_*n*, *m*_. It does not imply that this performance is high per se.

### Classifiers

In order to investigate whether the impact of variability is classifier specific, we employed a broad range of classifiers, being the nearest centroid (NC) classifier, *k*-Nearest Neighbors (*k*-NN) with *k *∈ {1, 3}, a Support Vector Machine (SVM) with a linear kernel (SVMlin) and radial basis function kernel (SVMrbf), and the Random Forest (RF) classifier. For descriptions of the individual methods, see [31,32]. The NC and *k*-NN used a cosine based distance function (see Additional File 3). All SVM results were obtained using the R package e1071 and for each feature set a grid search was performed to find the best hyperparameter values. Classification results for RF were obtained using the R package randomForest. Further details are presented in Additional File 3.

### Computing environment

Although our proposed protocol is conceptually quite simple, it is computationally demanding to obtain results, since for each dataset, preprocessing method, split and signature size, a classifier needs to be trained and validated. In addition, performing perturbation experiments for certain classifiers such as nearest neighbors can be time consuming as well. One benefit of our protocol is that it lends itself well to parallelization. In order to perform our computations we used a grid with over 1600 cores, divided over 206 Dell PowerEdge blade servers, each with 2 Intel XEON L5420 Quadcore CPU's, with 16GiB FDB Dual Rank memory. All computations were performed using R [27] and Bioconductor [28].

## Results

The aim of our work is to get a comprehensive overview of the impact of feature variability on microarray breast cancer classification. We will operate under the null hypothesis that preprocessing and perturbation variability have no effect on feature selection and classification. Under this null hypothesis we expect that for different preprocessing methods or for perturbed versions of a dataset we 1) typically select the same features, 2) obtain identical class assignments and as a consequence 3) obtain overlapping P-curves and 4) obtain D-curves that are flat and close to zero. In addition, we expect to 5) obtain S-curves that are flat and close to zero as well. We first report our results of studying the impact of perturbation and preprocessing variability on feature selection, before moving on to their influence on classification.

### Impact of feature variability on feature selection

In this paper we focus on compact gene signatures. Unfortunately, feature selection on high-dimensional datasets, like the ones associated with microarray-based expression profiling, is typically unstable as different subsets of samples frequently lead to the identification of different feature sets [6]. From a classification perspective, such a difference does not necessarily signal a problem, as long as the performances of the sets are similar, although from a biological perspective it makes reasoning about the data much more challenging.

It has been observed that the impact of preprocessing strategies on differential expression detection is high [20]. Note that feature selection strategies in microarray literature are often based on univariate ranking strategies, e.g. based on SNR-statistics or t-tests [3]. One would expect that genes that are strongly differentially expressed are also highly ranked by univariate selection procedures and hence that feature selection is also influenced by feature variability. In this section we show several examples of the influence of perturbation and preprocessing variability on signature composition, i.e. feature selection. For the Rosetta data it was not possible to assess the influence of preprocessing variability, as for this dataset only processed data is publicly available.

#### Van 't Veer breast cancer signature composition is sensitive to perturbation variability

As a first example, consider the feature selection step used to identify the 70-gene breast cancer signature by Van 't Veer et al. [2]. This signature is comprised of the top-70 genes with an absolute Pearson correlation coefficient with the class label (0 or 1) larger than 0.3 as obtained from the 78 training samples of Van 't Veer. Note that the computation of correlation coefficients can be very sensitive to the presence of outliers. To test the sensitivity of this feature selection step, we created 1000 perturbed instances of the training set, using the Rosetta uncertainty estimates (see Methods section and Additional File 3) and recomputed the Pearson correlation coefficients.

**Figure 2:**
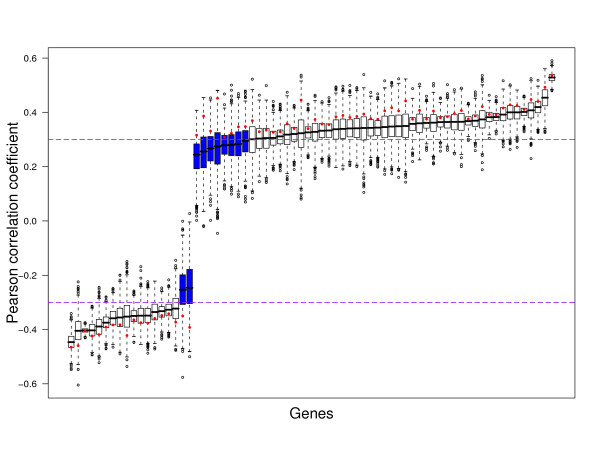
**Impact of perturbation variability on feature selection criterion of 70-gene signature**. Distributions are shown of the feature ranking criterion (Pearson correlation) calculated over 1000 perturbations of the 78 training samples of the Van 't Veer dataset. The dashed purple lines indicate the used absolute threshold of 0.3. Blue boxes indicate genes that do not meet this filter criterion in more than 50% of the perturbations. The red dots indicate the correlations obtained using the unperturbed expression values.

Figure 2 shows the sensitivity results of the feature selection step to perturbation variability. We see that perturbation generally weakens the correlation of a gene with the class label vector. This is reflected by the red points, which were always located in the tails of the distributions. We also see that the correlations of weaker genes sometimes shrink to zero, indicating that they lose the connection with the class label vector. Although most genes will still be selected for most perturbations, there are ten genes, indicated by blue boxes, that would not have been selected for the majority of the perturbed training sets. Furthermore, the ranges of the correlation coefficients for the genes are quite large, implying that rankings based on them are unstable, as in [6].

#### High impact of perturbation variability on feature rankings for Affymetrix datasets

In the previous example, the composition of the signature was given. In practice, however, the identification of a suitable set of marker genes is part of the discovery process. Our protocol, similarly to the protocol suggested in [3], employs a signal-to-noise ratio based ranking on each training split, in order to identify useful features for signature construction. This implies that the composition of our signatures is fully determined by the outcome of the ranking step and independent of the classifier used.

**Figure 3:**
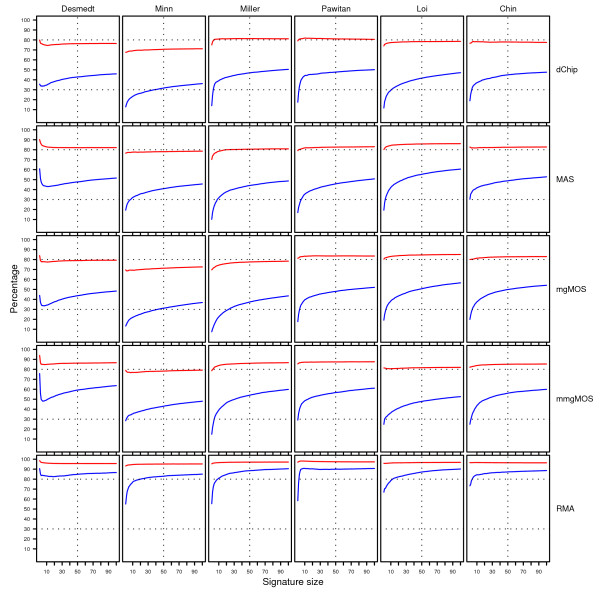
**Impact of perturbation variability on feature selection for the Affymetrix datasets**. Each dataset was split 50 times into a training and validation set, for which the validation set was subsequently discarded. Ranking was done only on the training sets. In addition, for each training set 50 perturbed versions were created and for each perturbation the overlap between *F*_*n*, *m*, *k *_and  and between  and *F*_2*n*, *m*, *k *_was determined, yielding 50·50·2 = 5000 overlap estimates for each list size *n*. The blue curves provide for each *n *∈ {1,...,100} the mean overlap taken over all corresponding estimates. The red curves indicate the associated average relative strengths between the feature sets *F*_*n*, *m*, *k *_and .

We examine the overlap between SNR-based rankings obtained using an unperturbed and a perturbed version of a dataset. Let *F*_*n*, *m*, *k *_denote the top-*n *ranked genes, using data from preprocessing method *m*, for split *k *and let  be the ranking obtained using a perturbed version. Although a complete overlap between these lists is preferable, we would at least hope to find a substantial part of the top half of one list in the other list. How large these parts are, is shown in Figure 3.

For most preprocessing methods the impact of perturbation noise appears to be large. Although the overlap increases when signature size increases, the overlap between a ranking based on unperturbed data and one based on perturbed expression data is generally less than 50%. In the Desmedt dataset there were two genes that almost always appeared at top of the SNR rankings in each split, which is the reason of the shape irregularity seen in the (blue) overlap curves for the study by Desmedt. For RMA, the overlap between rankings based on unperturbed and perturbed versions is much larger, with overlaps between 80 and 90%. In comparison to the other preprocessing methods RMA appears to give lower estimates on the measurement errors, although on the basis of our data one cannot tell if RMA underestimates the errors or if the other methods typically overestimate the errors.

Note that a lack in overlap does not necessarily signal a problem if the selected feature sets are of equal strength. Although the overlap for most preprocessing methods is quite low, the related relative strengths (see Methods section) are still high, with values of over 80% for most preprocessing schemes and values of over 95% for RMA, indicating that the performance for signatures based on the different rankings is expected to be comparable. A similar observation was made in [6], which for instance shows that on the Van 't Veer data the performance of the second best 70 genes was very comparable to the performance achieved by selecting the top-70 genes. Note that the latter two lists by construction have an overlap of zero. For some datasets many equally performing signatures exist, as was also noted in [33].

#### Affymetrix breast cancer signature composition is sensitive to preprocessing variability

**Figure 4:**
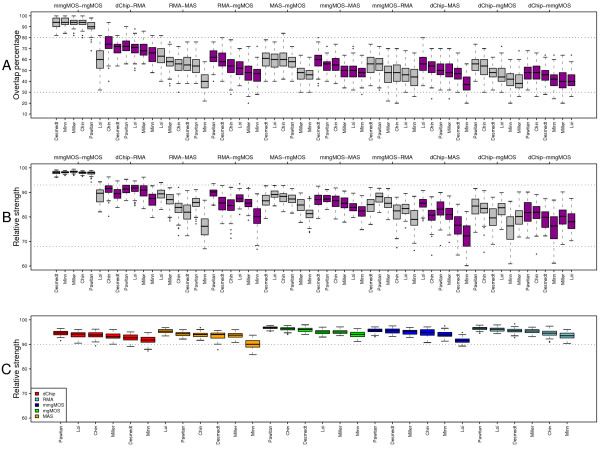
**Impact of preprocessing variability on feature selection for the Affymetrix datasets**. Comparison of top-100 ranked features lists *F*_100, *m*, *k *_and *F*_100, *m'*, *k*_, as obtained using different preprocessing strategies *m *and *m'*, for different splits *k*. A) Percentage of the top-half of one list that is found in the other list, and vice vera. Each boxplot represents the distribution of such percentages over 50 splits, for a specific pair (*m, m'*) (indicated on top of the figure). For each split, we determine the percentage of *F*_50, *m*, *k *_found in *F*_100, *m'*, *k *_and the percentage of *F*_50, *m'*, *k *_found in *F*_100, *m*, *k*_. Each distribution thus contains 50·2 = 100 points. All boxplots corresponding to the same preprocessing pair are colored similarly. In total there are 15 distinct pairs. The pairs are ordered by the observed median overlap over all six datasets. B) Distributions of the relative strength scores for top-ranked feature lists corresponding to the various preprocessing pairs. C) Relative strength of the top-100 multi-ranked gene lists with respect to the original rankings, for each preprocessing method and each Affymetrix dataset.

Here we inspect the overlap between top-ranked feature lists, as obtained using different preprocessing methods i.e. we consider preprocessing variability. Consider two top-ranked feature lists, based on two different preprocessing schemes *m *and *m'*, say of size 100, i.e. *F*_100, *m *_and *F*_100, *m'*_. Similarly to the example in the previous section, we would hope to find a substantial part of the top half of one list in the other list. Figure 4A shows the overlap of the top-50 of one list in the top-100 of the other.

Different preprocessing strategies give rise to the selection of different features as well, as for all preprocessing pairs again none have a complete overlap. Within the same preprocessing family, i.e. mgMOS and mmgMOS, the overlap is high, although for the dataset of Loi there is already quite a discrepancy. For the remaining pairs we see that the overlap between top-ranked feature lists can be quite low. The overlap between different preprocessing families for the various datasets lies between 30 and 80%. The highest overlap between methods from different families was found between rankings based on dChip and RMA, with a median overlap of 70% over six datasets. The overlap between RMA and MAS is lower, with a median of only 56% over all six datasets. From the last block, we can see that even though dChip and mmgMOS are both multi-chip preprocessing strategies, they usually tend to pick different feature sets, with a median of 44% over six studies. Excluding the (mgMOS, mmgMOS) pair, the median overlap over all data sets and splits is 52%. Note that this lack in overlap is completely due to the preprocessing method chosen, as the feature selection criterion, the array platform, and the set of samples (and hence the sample handling and hybridization conditions) are all identical. Comparing the overlap from Figure 4A (preprocessing variability) to that in Figure 3 (perturbation variability) we see that the scores have a similar range, i.e. around 50%.

#### Relative strength of Affymetrix-based breast cancer signatures is more robust against preprocessing variability

In the previous section, we saw that the use of a different preprocessing strategy typically leads to the identification of a different feature set and that the overlap between top-ranked feature sets for different preprocessing pairs can be quite poor. Figure 4B shows the distribution of the relative strengths for top-ranked feature lists from the example in Figure 4A. The order of the boxplots in panel B is the same as in panel A. Comparing the two panels, we see that a lower overlap is typically associated with a lower relative strength as well. However, although the overlap between top-ranked features sets can be quite poor, the relative strengths are reasonably high. The highest scores are again obtained between preprocessing pairs from the same family. Since the (m)mgMOS models have a large overlap in top-ranked lists, their relative strengths are high as well, with values of over 90%. Even for the Loi dataset, the median relative strength over 50 splits is still above 89%, while the actual overlap is quite poor with a median of 60%. Furthermore, distributions of relative strengths for the Minn dataset, for pairs of preprocessing strategies from different families (all blocks except the first one), are mostly wider and have a lower tail than the other distributions. This is probably caused by the small number of samples in the Minn dataset. Comparing the relative strengths from Figure 4B (preprocessing variability) to those in Figure 3 (perturbation variability) we see that the scores are similar, with a mean relative strength of 85.1% taken over all entries in Figure 4B to a mean relative strength of 84.2% taken over all entries corresponding to Figure 3 at *n *= 100.

#### Preprocessing-neutral top gene lists

The lack of overlap between top-ranked lists corresponding to different preprocessing methods, as observed in Figure 4A, presents an additional complication in comparing performances between signatures based on such lists as we then cannot know whether a difference in performance is due to a difference in selected features, or due to a difference in feature values as obtained from the preprocessing method. In order to compare the performances of signatures constructed on data from different methods, ideally we would like to use the same set of features. Here, we show that we can obtain a ranking with a high relative strength over all preprocessing methods by combining the ranking information associated with the different preprocessing methods. In the previous sections, each top-ranked feature set was based on data from a single preprocessing method. For a given method *m *∈ *M *we will refer to this ranking as a *single-rank *feature list. The strength of a feature *i *for method *m*, denoted by *S*_*m*_(*i*), was measured by SNR_*i*, *m*_. Here we base the strength of feature *i *on the average of the individual strengths, as obtained by the different preprocessing methods in *M*, i.e., we use a strength(4)

In the remainder we will refer to the ranking based on this combined strength *S*(*i*) as a *multi-rank *list. For each split *k *and for each dataset, we computed the top-100 ranked feature list based on this multi-rank strategy and determined its relative strength in the top-rank list *F*_100, *m*, *k *_for each preprocessing method *m*. Figure 4C gives for each dataset the distribution of these relative strengths. Relative strengths of the multi-ranked lists are high, with a median score of over 90% for all datasets. In order to decouple the effect of feature selection from the impact of perturbation and preprocessing variability on classification performance, we will therefore mainly use multi-rank gene lists, although all experiments on the Affymetrix datasets were also performed using the single-rank lists.

### Impact of feature variability on classification

We start our investigation of the effects of feature variability on classification by taking an in-depth look into the Van 't Veer [2] and van de Vijver [34] expression data, which is based on the Rosetta error model. Starting from a single split of the data and using only features as considered in the original publications, we progress towards a more sophisticated setting, ending up in using the full sensitivity analysis protocol and applying it on all Affymetrix datasets using multiple preprocessing strategies and multiple classifiers.

#### Van 't Veer signature is sensitive to perturbation variability

We investigated the classification stability of the original 70-gene signature of Van 't Veer et al. [2]. The classifier used for the construction of their signature is a nearest centroid classifier. Classification for this classifier can be linked to a *discriminant score *(see Additional File 3), by which we assign a sample to the good prognosis class if the discriminant score is positive, and to the poor prognosis class otherwise. We use the original Van 't Veer training set of 78 samples to estimate the class centroids. As a validation set we took the 106 remaining samples in the Rosetta dataset. Next, using the uncertainty information estimated by the Rosetta error model (see Method section and Additional File 3), we created 1000 perturbed versions of the validation set and classified these with the classifier built on the original training data.

**Figure 5:**
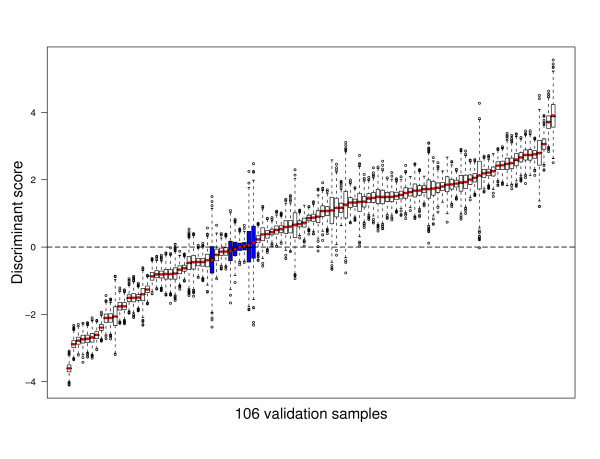
**Impact of perturbation variability on discriminant score**. Distributions are shown of the discriminant score **x**^*T*^**w **for each of the 106 validation samples of the Rosetta dataset, when using a nearest centroid classifier built on the 70-gene profile of [2], over 1000 perturbations. Perturbed expression data is based on the Rosetta error model. Red dots indicate the discriminant scores corresponding to the unperturbed expression data. The blue boxes indicate samples with a map-score of at least 25%.

Figure 5 shows the impact of perturbation variability on the discriminant scores for each of the 106 cases. Note that a validation sample is stably classified if the discriminant score is either positive for all its perturbed instances, or negative for all its perturbed instances. For some samples the variation of the corresponding discriminant score is small, while for others it is quite large, reflecting the fact that measurements for the same probe on different arrays are associated with different measurement errors. In addition, the individual distributions are quite symmetric, which stems from the fact that the classifier is linear and we added symmetrical noise. Perturbation variability can indeed disrupt the classification process, since for seven samples (indicated in blue) the box-and-whisker plots cross the horizontal threshold line at height zero. Note that the boxes in a box-and-whisker plot indicate the interquartile range of a distribution and thus these seven samples have an associated map-score of at least 25%.

#### A map-matrix example for the Rosetta dataset

Now we extend the example of the previous section by considering a sequence of 100 signatures constructed using the top-100 ranked features from the Van 't Veer data and zoom in on the impact of perturbation variability on classifications of individual samples from the Rosetta data by taking an in-depth look at a map-matrix, such as the ones obtained from our sensitivity analysis protocol. Classifications are again performed using the nearest centroid classifier. The *n*^*th *^signature is constructed using only the top-*n *features. Note that this setting is similar to our protocol, in which at step 2 we take the 78 training cases of Van 't Veer data as a training set, the 106 remaining samples as a validation set, at step 3 take the top-100 features as described above and at step 4 train a sequence of 100 NC classifiers, thus yielding 100 signatures. Following the protocol, at step 7 we obtain a map-matrix, which in this case is a 106 by 100 matrix, where the entry at row *k *and column *n *contains the map-score of sample *k *using a signature involving the top-*n *features.

**Figure 6:**
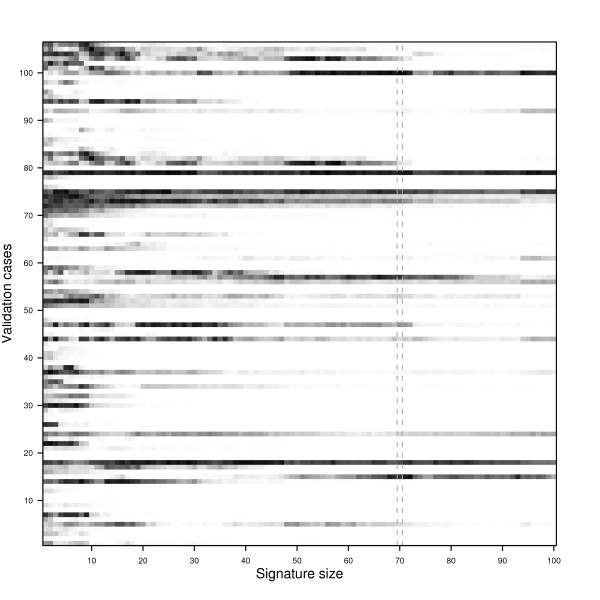
**A map-matrix example for the Rosetta dataset**. The minimum assignment percentages (white = 0%, black = 50%) for the 106 validation samples and signatures of increasing size, determined over 1000 perturbations of the validation data. The column indicated by the dashed lines corresponds to the original 70-gene signature.

Figure 6 visualizes the map-matrix of this example by means of a heatmap. Here white entries indicate completely stable assignments, i.e. the map-score is zero, while black entries indicate random class assignments. From the figure we see more dark areas on the left than on the right, indicating that classification is generally less stable if fewer genes are used. In addition, for very small signature sizes i.e. less than 10, the classification of virtually all samples can be disrupted by perturbation variability, as almost none of the corresponding cells are completely white. Furthermore, we observe that for some samples, adding features may first reduce the impact of variability, whereas adding more features later increases the impact of variability again and vice versa. This may be due to the fact that either features are added that are quite noisy for such a sample, or that such features draw these samples closer to the decision boundary. Finally, even for large signatures the classification of some samples can still be affected by perturbation variability, although the number of such cases is typically low.

#### Performance and stability curves for the Rosetta dataset

**Figure 7:**
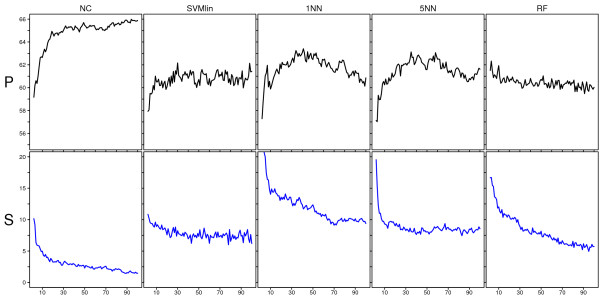
**Performance and stability curves for the Rosetta dataset**. P and S-curves for the Rosetta data for various classifiers. The *x*-axis shows the signature size, the *y*-axis in the upper panel gives the average balanced accuracy over 50 splits and the *y*-axis in the lower panel gives the average percentage of cases over 50 splits with a map-score larger than 35. Each column shows the results for a different classifier.

In the previous section, results were obtained using only a single split of the data in a training and validation set. Here we apply the full sensitivity analysis protocol to the Rosetta dataset consisting of 184 samples. Figure 7 shows the resulting performance (P) and stability (S) curves for five classifiers, based on 50 splits of the data. The NC classifier performs best and clearly increases its performance when using more features with a highest performance of around 65%. This is comparable to the estimates reported in [3-5]. Furthermore, on this dataset the NC classifier also had the best S-curve. S-curves generally improve when using more features, however, none are flat and close to zero, indicating that perturbation variability can consistently disrupt these classifications. For the NC classifier the impact of perturbation variability on this dataset quickly diminishes, with an average number of unstable assignments leveling off around only 2.5% at a signature size of 100. For other classifiers we see that the impact of perturbation variability is higher than for the NC classifier and especially the 1-nearest neighbor seemed very sensitive at small signature sizes, only leveling off around 10% at a size of 100 features. Although stability is a desirable characteristic, we should not directly link ascending P-curves to descending S-curves and simply attribute the higher performance of the NC classifier to perceived noise tolerance. Although the S-curves typically decrease when the signature size increases, the P-curve does not generally show such a monotonic behavior. For instance, the nearest neighbor classifier shows a decreasing P-curve for larger signature sizes and is indeed known to be intolerant to the inclusion of irrelevant features.

#### Impact of feature variability for Affymetrix datasets

In order to investigate the impact of both preprocessing variability and perturbation variability on Affymetrix GeneChip data, we ran our complete protocol, for each of the six Affymetrix datasets, five different preprocessing methods (mgMOS, mmgMOS, MAS5.0, dChip, and RMA), and six classifiers (NC, 1NN, 3NN, SVMlin, SVMrbf, and RF). Each dataset was analyzed with 50 different splits into a training and validation set. Each validation set was perturbed 1000 times in order to infer the S-curves. Furthermore, the experiments were performed using both the single-rank and multi-rank sets.

**Figure 8:**
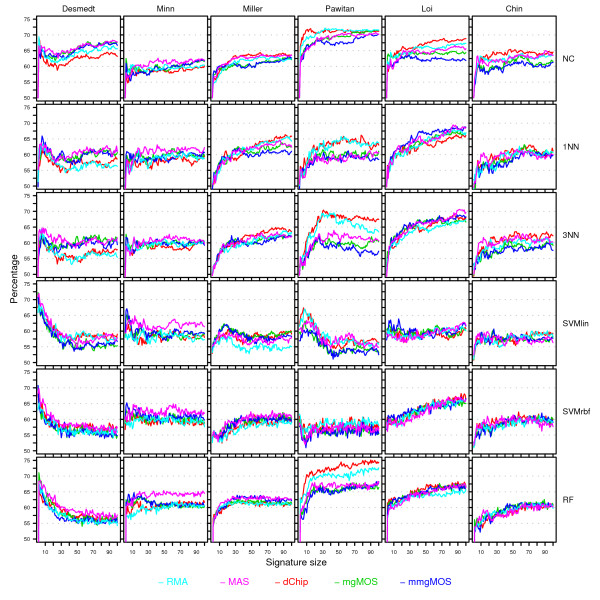
**Performance curves for the Affymetrix datasets**. Rows represent curves obtained using different classifiers, while columns represent curves for different datasets. Within each cell, performance curves associated with different preprocessing methods are shown in separate colors. The color scheme is shown at the bottom of the figure. Within a cell the *x*-axis provides the signature size, while the *y*-axis gives the average balanced accuracy over 50 splits. For each dataset and split, the top-100 feature set was computed using the multi-rank strategy and this ranking was subsequently used for all classifiers in order to construct signatures.

**Figure 9:**
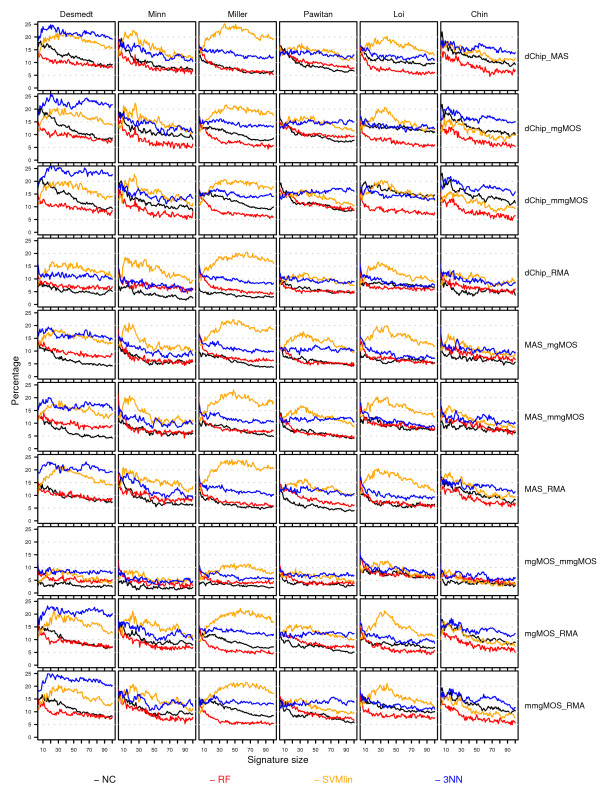
**Discordance curves for the Affymetrix datasets**. Rows represent different preprocessing pairs, while columns represent curves for different datasets. Within each cell, discordance curves corresponding to different classifiers are shown in separate colors. The color scheme is shown at the bottom of the figure. Within a cell the *x*-axis provides the signature size, while the *y*-axis gives the average percentage of cases, over 50 splits, of inconsistent class assignments on the unperturbed validation sets. For each dataset and split, the top-100 feature set was computed using the multi-rank strategy and this ranking was subsequently used for all classifiers in order to construct signatures.

**Figure 10:**
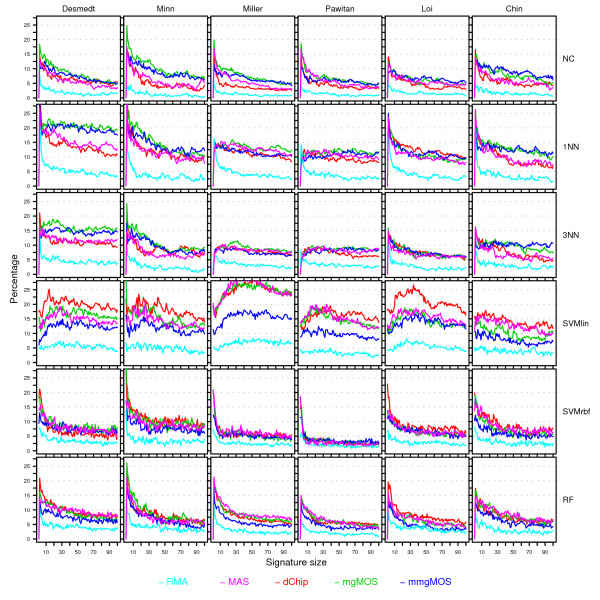
**Stability curves for the Affymetrix datasets**. Rows represent curves obtained using different classifiers, while columns represent curves for different datasets. Within each cell, stability curves associated with different preprocessing methods are shown in separate colors. The color scheme is shown at the bottom of the figure. Within a cell the *x*-axis provides the signature size, while the *y*-axis gives the average percentage of cases over 50 splits with a map-score larger than 35. For each dataset and split, the top-100 feature set was computed using the multi-rank strategy and this ranking was subsequently used for all classifiers in order to construct signatures.

From Figure 4A we saw that different preprocessing methods tend to pick different features and that the overlap between rankings can be low. Hence, if we use single-rank sets, we will observe a combined effect where differences between curves corresponding to different preprocessing methods can be due to a difference in signature composition, as well as due to feature variability. The advantage of the multi-rank approach is that for a given dataset-classifier pair, observed differences in performance, discordance (D, see Methods section), and stability curves are not due to a difference in signature composition, but solely due to feature variability. Using a signature based on a multi-rank set effectively decouples the impact of feature selection from the effect of feature variability on classification performance. Given the high relative strengths of the multi-rank sets, as observed in Figure 4C, we therefore show in the main text only the figures corresponding to these multi-rank sets. Figures 8, 9 and 10 show the resulting P, D and S-curves, respectively, for all 36 classifier-dataset combinations. The corresponding P, D and S-curves for the single-rank sets are shown in Additional Files 4, 5 and 6, respectively.

#### Lack of overlap in performance curves on Affymetrix datasets

Figure 8 shows the P-curves for the multi-rank based experiments. Note that we are less concerned with the actual shape of the performance curves, but we are mainly interested if different preprocessing methods lead to overlapping curves. For most dataset-classifier pairs the corresponding P-curves indeed show the same trends. In direct contradiction to our null hypothesis, however, several large deviations can be seen, most notably on the Pawitan dataset for multiple classifiers (NC, 1NN, 3NN, RF). On this dataset there seems a clear advantage in using dChip or RMA expression estimates. Although RMA usually performs well, it does not consistently give the best performance curves. In fact, no preprocessing method is clearly superior to all other methods. On the datasets of Desmedt and Minn, for instance, MAS often outperforms both RMA and dChip. On most datasets balanced accuracy rates between 60 and 75% could be achieved, depending on the classifiers and signature size. In most cases the performance increases for larger sized signatures, although on the dataset of Desmedt high accuracies could be achieved using only a few features. Although certain preprocessing-classifier pairs have a good performance for some datasets, such performance advantages cannot be maintained on the other datasets. When comparing classifiers, we see that simple classification models like the NC classifier and the NN classifiers typically perform at least as well as more complex classifiers like SVM or RF. Similar observations on the performance of simple versus more complex classifiers in the context of microarray data have been made in [3,4,35]. In our experiments the SVM classifiers did not perform well. For instance, even though SVMlin and NC are both linear classifiers, the NC classifier is clearly superior. Although a large grid of hyperparameter values was attempted for SVM, it proved hard to find the correct hyperparameter values.

#### Different preprocessing methods produce discordant class assignments

In the previous section we observed that in several studies there was a lack in overlap between performance curves for different preprocessing methods, clearly indicating a discordance in outcome prediction. Even in the case of overlapping performance curves, however, one cannot ascertain that the individual class assignments are concordant. Figure 9 shows for several classifiers (NC, 3NN, SVMlin, RF) the discordance curves corresponding to the P-curves of Figure 8. For all preprocessing pairs clear discrepancies can be seen, which is in direct disagreement with our null hypothesis. The highest D-curves for the selected classifiers are observed for the 3NN and SVMlin classifier, with an overall median discordance (over all signature sizes and splits) of 12.6% and 14.2%, respectively. The NC and RF classifiers show lower numbers of discordant class assignments with an overall median discordance of 8.4% and 7.5%, respectively. For the latter two classifiers the discordance also clearly decreases with larger signature sizes, leveling off at a signature size of 100 with an overall median discordance of 6.8% and 6.4%, respectively. The discordance is often larger in the poor prognosis group than in the good prognosis group (see Additional Files 7 and 8, respectively). Note that in most breast cancer datasets, the former group is also much smaller than the latter. When using balanced performance indicators, a discordance in the poor prognosis group is then more heavily penalized than a discordance in the good prognosis group. For instance, in Figure 8 a clear difference in performance curves can be seen for the preprocessing pair (dChip, MAS), when applying the RF classifier on the Pawitan dataset. From Figure 9, however, the lack in concordance for the preprocessing pair (dChip, MAS) does not seem much larger than on other datasets. From Additional File 7, we can see that for this preprocessing pair and dataset the number of discordant cases for the RF classifier in the poor prognosis group is indeed higher, with an overall median of 18.7% compared to an overall median of 8.5% on the remaining datasets.

#### High impact of perturbation variability for small signature sizes on Affymetrix datasets

Figure 10 shows the stability curves associated with the class assignments of Figure 8. None of the S-curves are flat and located near zero, which is again in direct contradiction with our null hypothesis. For most classifiers and preprocessing methods the impact of perturbation variability is high at small signature sizes, in which over 10% of the assignments are unstable. Similarly to Figure 7, the impact of perturbation variability quickly diminishes for increasing signature sizes, although for most classifiers approximately 5% of the assignments are still unstable at a signature size of 100. The perturbations corresponding to RMA appear to be smaller compared to those of the other preprocessing methods, as RMA consistently gives the lowest S-curves. These S-curves cannot always be associated with the best P-curves though. When comparing classifiers we see that the impact of perturbation variability can be quite different for different classifiers. Certain classifiers like SVMs [36] and RF [32] have been claimed to be noise tolerant. We did not find clear evidence that SVM or RF are more tolerant to the types of perturbation variability as discussed here. Although the SVMrbf indeed appears very stable on some datasets, its performance is also very poor compared to other models (Figure 8). The S-curves corresponding to SVMlin are notably different and the class assignments seem particulary sensitive to perturbation variability. No satisfactory answer was found that could explain this observed behavior. Furthermore, in our experiment the RF classifier is not more noise tolerant than for instance the NC classifier. For small signature sizes, i.e. fewer than 10 genes, the average number of unstable assignments (taken over all studies and all preprocessing methods except RMA) is 11.8% for RF, compared to only 10.1% for the NC classifier. At a size of 100, the average number of unstable assignments for RF and NC is 5.3% and 4.6%, respectively. Finally, the impact of perturbation variability for the nearest neighbor classifiers appears to be larger. For 1NN and 3NN the average number of unstable assignments at signature sizes less than 10 is 15.5% and 11.3%, respectively, and at size 100 it is 10.6% and 8.8%, respectively.

## Discussion

Finding high-quality stable biomarkers in breast cancer applications using microarray expression profiling has proven to be quite challenging with reported balanced accuracy rates for most breast cancer signatures somewhere between 60 and 70%. Signature composition strongly depends on the subset of patient samples used for feature selection [6]. Furthermore, a high level of inconsistency between individual class assignments between different signatures has recently been reported [7]. Differences in array platforms as well as biological variability have been conjectured to play a major role in these discrepancies.

We designed an experimental protocol to evaluate the impact of two other types of variability, namely preprocessing and perturbation variability, on signature composition and classification. For this purpose several state of the art and frequently applied preprocessing methods were selected. Complementary to Ein-Dor et al. [6], we showed that signature composition is strongly influenced by perturbation variability and preprocessing variability, even if the array platform and the stratification of patient samples are identical. In addition, using our multi-rank feature sets we showed that there is often a high level of discordance between individual class assignments for signatures constructed on data coming from different preprocessing schemes, even if the actual signature composition is identical. For the single-rank feature sets, the observed discrepancies were even larger. No preprocessing scheme, however, yielded data that was clearly superior for classification purposes. When comparing preprocessing variability to perturbation variability, we found their impact on feature selection to be equally strong. On classification, however, the impact of preprocessing variability often remained strong with increasing signature size, whereas the impact of perturbation variability quickly diminished.

Preprocessing noise is mainly caused by different underlying assumptions that are made on the data and on the available sources of information that are used. Some methods deliberately ignore some sources of information or exclude certain steps. RMA, for instance, does not use mismatch probe information to infer expression levels, while standard applications of dChip do not perform a background correction step. Note that the latter can have great implications on the final expression data for both one [37] and two-color array data [38].

Our stability analysis performs explicit noise tolerance tests for a diverse set of classification routines, by using the class assignments of perturbed expression profiles. The results indicate that all classifiers considered were sensitive to perturbation variability, although the impact was much stronger at small signature sizes and quickly diminished for larger signature sizes. Furthermore, in most cases we found the level of noise tolerance for the NC, SVMrbf, and RF classifiers to be very comparable.

We chose to use realistic estimates of gene-wise measurement error in the stability analyses. Methods like the Rosetta error model, but also the mgMOS and mmgMOS models, are specifically designed to obtain such uncertainty information associated with the fitted expression data. Methods like dChip, RMA, and MAS 5.0 are not designed with this goal in mind. However, some uncertainty information can be derived from the summarization step, as performed in the preprocessing cascade. Although the uncertainty estimates for dChip, RMA and MAS are based on the same type of information, we found that perturbations corresponding to RMA seemed much less severe than those based on other methods; cf [21]. For the Affymetrix preprocessing methods a potential problem with basing uncertainty estimates solely on the summarization step is that most probesets consist of a small number of probes, with a median size of 11 for the GeneChips used here, which can make the standard error estimates less reliable. Although the stability curves for MAS and dChip were closer to those of the mgMOS and mmgMOS models, from our experiments one cannot tell if RMA underestimates the errors or if the other methods overestimate the errors.

**Figure 11:**
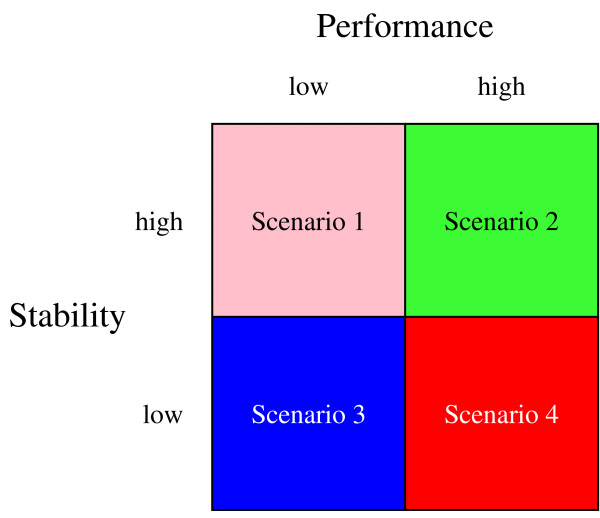
**Trade-off dilemma of performance versus stability**. Different scenarios are shown for the performance of a classifier versus its stability. Scenario 1: Stable yet poor performance, always achievable by a decision rule that assigns all samples to the same class; Scenario 2: Preferred scenario; Scenario 3: Random classifier; Scenario 4: Unrealistic perturbations, likely to happen when using jitter.

Our results also show that a high stability and a good performance do not always go hand in hand (Figures 8 and 10). Although stability is a desirable property, it is sometimes conflicting with achieving a high performance, which presents us with a dilemma, similar to the bias-variance dilemma [31]. To this end, consider Figure 11. From a classification standpoint, the second scenario is obviously the preferred scenario, while scenario three is equal to tossing a coin. Note that scenario one can always be achieved by using a rule that assigns all samples to the same class. Such a rule is extremely stable, yet when using balanced accuracy rates, will also have a poor performance. This scenario was sometimes observed for the SVM classifiers. For both linear and non-linear SVMs, parameter estimation was hard. This might be an explanation for the observed poor performance, although our performance estimates on single datasets for SVM were often comparable to those reported earlier [4,26]. Finally, scenario four would be a strong indicator that the perturbed expression profiles are not very realistic, given the fact that performance and stability are both measured on the same validation data. This scenario was, however, not observed in our experiments. We did encounter this scenario in attempts to base perturbed expression profiles on jitter i.e. artificial noise estimates. The main problem in using jitter is that such estimates are either much too low or much too high and therefore this type of perturbation was not further pursued here.

Note that our goal was not to compare classifiers or even to find optimal biomarkers per se and it is likely that the performance of some classifiers can be further improved e.g. by changing the feature selection step in our protocol, which in our case was based on univariate signal-to-noise-ratio statistics. Alternative univariate ranking strategies such as those based on the t-test, Mann-Whitney u-test, and Mahalanobis distance were reported to perform similarly [3] and were therefore not pursued here. Note that the former methods all construct rankings based on binary class-label information. Survival information on which the class labels are based could be incorporated in the ranking step as well. For instance, in [39] a 76-breast cancer gene signature was derived using a ranking step based on information from univariate Cox proportional-hazards regression models using the length of distant metastasis free survival.

For some classifiers it might be advantageous to resort to multivariate wrapper-based feature selection methods. Perhaps the simplest computationally efficient multivariate wrapper is the Top-Scoring-Pair (TSP) classifier [40], which performs its classifications on the basis of the expression values of just two genes. On several classical tumor data sets e.g. leukemia [41], colon [42], lymphoma [43], and prostate [44], the TSP was able to obtain balanced accuracy rates well over 90%; see [45]. In [40] the TSP is claimed to be invariant to pre-processing changes, as it is invariant to any monotonic transformation of the expression data. Although our forms of feature variability are very realistic, they can certainly not be considered as monotonic transformations of the raw expression data. Initial experiments with the TSP on the Affymetrix breast cancer datasets revealed that the classifier was extremely sensitive to feature variability, with corresponding balanced accuracy rates often close to 50% (data not shown). On the Rosetta data a similar observation on the performance of the TSP was reported in [46]. As the TSP uses only two genes, these results are in agreement with our observation that breast cancer signatures comprised of few genes seem very susceptible to feature variability. In addition, in [46] several alternative multivariate approaches were benchmarked, with the main conclusion that multivariate variate selection approaches often do not lead to consistently better results than univariate approaches. Moreover, compared to multivariate approaches, univariate ranking procedures have the benefit of a considerable computational speed up, which in our case was very important considering the large number of experiments performed.

Our sensitivity analysis was performed on a sizable collection of patient sample hybridizations and in a breast cancer classification context, which is different from the small scale spike-in and dilution studies on which most previous microarray sensitivity analyses were performed [47,48]. One advantage of the latter two types of studies is that the ground truth is known, which for most breast cancer studies is less obvious. In our framework, however, under the null hypothesis we also know exactly what should be expected, i.e. for different preprocessing methods or for perturbed versions of a dataset we should have selected the same features, had overlapping P-curves and obtained D-curves and S-curves that were zero for all signature sizes, as stated in our null hypothesis. Based on the outcome of our experiments, however, we conclude that this is not the case, and hence we conclude that in microarray *breast cancer *studies feature variability can have a strong impact on both feature selection and classification. We conjecture feature variability to be less of an issue in microarray studies for which a high performance can be obtained such as for the classical tumor datasets mentioned above. Note that these studies all deal with tissue-type recognition problems, which are considerably easier classification problems than event prediction studies, such as the breast cancer studies treated here; see also [3].

Finally, the focus of this paper has been of a descriptive nature, analyzing the impact of feature variability. Obviously, one would next like to enhance the performance and stability of classifiers by exploiting the feature variability information. For instance, in the context of point injection techniques, one can use the perturbed expression profiles as additional candidates to be injected, instead of the rather artificial candidates obtained by linear interpolation [35]. Another avenue that one may take is to directly increase classification concordance by explicitly enforcing it, for instance in a wrapper framework.

## Conclusion

We performed an extensive sensitivity analysis of microarray breast cancer classification under feature variability. Our results indicate that signature composition is strongly influenced by preprocessing variability and perturbation variability, even if the array platform and the stratification of patient samples are identical. In addition, we show that there is often a high level of discordance between individual class assignments for signatures constructed on data coming from different preprocessing schemes, even if the actual signature composition is identical.

We presented evidence of discrepancies induced by technical variation that cannot be considered negligible, as previously claimed by some researchers [17]. We therefore strongly recommend that feature variability is taken into account during the construction of a signature, especially when using microarray technology for the classification of individual patients. In addition, measures should be taken to minimize the technical variation of microarray procedures when used for such high impact applications as cancer diagnostics.

## Authors' contributions

HMJS drafted the manuscript, designed the experiments, created the software implementations and finalized the manuscript. PDM co-designed the experiments, helped develop the software implementations, and drafted the manuscript. RvdH provided expert advice on microarray technology and helped draft the manuscript. MJTR critically reviewed the manuscript. WFJV instigated and guided the research project, co-designed the experiments and helped draft the manuscript. All authors read and approved the final manuscript.

## Supplementary Material

Additional file 1 **Overview of 947 Affymetrix hybridizations**. The column *DataSetName *indicates to what study each hybridization corresponds. For each study the repository and corresponding accession number can be found in Table 1 in the main text. The column *FileName *indicates the exact file name for each hybridization as used in the corresponding repository. For the datasets of Desmedt, Minn, Loi and Chin, the class label was based on the time of distant metastasis free survival (*t.dmfs*, in months) and corresponding event indicator *e.dmfs*. For the datasets of Miller and Pawitan, the class label was based on the time of breast cancer specific overall survival (*t.sos*, in months) and corresponding event indicator *e.sos*. Click here for file

Additional file 2 **Overview of additional 87 hybridizations from the Van de Vijver**. The column *SampleID *indicates the identifiers as used by Van de Vijver et al. [34]. The selected hybridizations represent all lymph-node negative cases that were not yet contained in the original publication by Van 't Veer et al. [2]. Click here for file

Additional file 3 **Supplementary information on perturbation schemes and classifiers**. The file contains additional information on how to construct perturbed expression profiles for MAS5.0, dChip and the Rosetta data. In addition, information on parameter settings for the SVM and RF classifiers is provided. Click here for file

Additional file 4 **Performance curves for the Affymetrix datasets using the single-rank feature sets**. Rows represent different preprocessing pairs, while columns represent curves for different datasets. Within each cell, performance curves corresponding to different classifiers are shown in separate colors. The color scheme is shown at the bottom of the figure. Within a cell the *x*-axis provides the signature size, while the *y*-axis gives the average balanced accuracy over 50 splits. For each dataset and split, the top-100 feature set was computed using the single-rank strategy and this ranking was subsequently used for all classifiers in order to construct signatures. Click here for file

Additional file 5 **Discordance curves for the Affymetrix datasets using the single-rank feature sets**. Rows represent different preprocessing pairs, while columns represent curves for different datasets. Within each cell, discordance curves corresponding to different classifiers are shown in separate colors. The color scheme is shown at the bottom of the figure. Within a cell the *x*-axis provides the signature size, while the *y*-axis gives the average percentage of cases, over 50 splits, of inconsistent class assignments on the unperturbed validation sets. For each dataset and split, the top-100 feature set was computed using the single-rank strategy and this ranking was subsequently used for all classifiers in order to construct signatures. Click here for file

Additional file 6 **Stability curves for the Affymetrix datasets using the single-rank feature sets**. Rows represent curves obtained using different classifiers, while columns represent curves for different datasets. Within each cell, stability curves associated with different preprocessing methods are shown in separate colors. The color scheme is shown at the bottom of the figure. Within a cell the *x*-axis provides the signature size, while the *y*-axis gives the average percentage of cases over 50 splits with a map-score larger than 35. For each dataset and split, the top-100 feature set was computed using the single-rank strategy and this ranking was subsequently used for all classifiers in order to construct signatures. Click here for file

Additional file 7 **Discordance curves for the poor prognosis cases in the Affymetrix datasets using the multi-rank feature sets**. Rows represent different preprocessing pairs, while columns represent curves for different datasets. Within each cell, discordance curves corresponding to different classifiers are shown in separate colors. The color scheme is shown at the bottom of the figure. Within a cell the *x*-axis provides the signature size, while the *y*-axis gives the average percentage of cases, over 50 splits, of inconsistent class assignments on the unperturbed validation sets. For each dataset and split, the top-100 feature set was computed using the multi-rank strategy and this ranking was subsequently used for all classifiers in order to construct signatures. Click here for file

Additional file 8 **Discordance curves for the good prognosis cases in the Affymetrix datasets using the multi-rank feature sets**. Rows represent different preprocessing pairs, while columns represent curves for different datasets. Within each cell, discordance curves corresponding to different classifiers are shown in separate colors. The color scheme is shown at the bottom of the figure. Within a cell the *x*-axis provides the signature size, while the *y*-axis gives the average percentage of cases, over 50 splits, of inconsistent class assignments on the unperturbed validation sets. For each dataset and split, the top-100 feature set was computed using the multi-rank strategy and this ranking was subsequently used for all classifiers in order to construct signatures. Click here for file
