# Supplementary material for: A comprehensive sensitivity analysis of microarray breast cancer classification under feature variability
Source: BMC Bioinformatics. 2009 Nov 26;10:389. doi: 10.1186/1471-2105-10-389 (PMC2789744; doi:10.1186/1471-2105-10-389)
Supplement: Additional file 1 — Overview of 947 Affymetrix hybridizations. The column DataSetName indicates to what study each hybridization corresponds. For each study the repository and corresponding accession number can be found in Table 1 in the main text. The column FileName indicates the exact file name for each hybridization as used in the corresponding repository. For the datasets of Desmedt, Minn, Loi and Chin, the class label was based on the time of distant metastasis free survival (t.dmfs, in months) and corresponding event indicator e.dmfs. For the datasets of Miller and Pawitan, the class label was based on the time of breast cancer specific overall survival (t.sos, in months) and corresponding event indicator e.sos. [file 1471-2105-10-389-S1.PDF]

Count	DataSetName	ClassLabel	FileName	t.dmfs	e.dmfs	t.sos	e.sos
1	Desmedt	Poor	GSM177885.cel.gz	23.32	1	-	-
2	Desmedt	Good	GSM177886.cel.gz	212.61	0	-	-
3	Desmedt	Poor	GSM177887.cel.gz	16.9	1	-	-
4	Desmedt	-	GSM177888.cel.gz	201.77	1	-	-
5	Desmedt	-	GSM177889.cel.gz	123.29	1	-	-
6	Desmedt	Good	GSM177890.cel.gz	209.9	0	-	-
7	Desmedt	Good	GSM177891.cel.gz	191.84	0	-	-
8	Desmedt	-	GSM177892.cel.gz	187.61	1	-	-
9	Desmedt	Good	GSM177893.cel.gz	193.77	0	-	-
10	Desmedt	Poor	GSM177894.cel.gz	39.77	1	-	-
11	Desmedt	Poor	GSM177895.cel.gz	36.65	1	-	-
12	Desmedt	Good	GSM177896.cel.gz	193.03	0	-	-
13	Desmedt	Good	GSM177897.cel.gz	201.58	0	-	-
14	Desmedt	-	GSM177898.cel.gz	131.16	1	-	-
15	Desmedt	Poor	GSM177899.cel.gz	22.26	1	-	-
16	Desmedt	Poor	GSM177900.cel.gz	32.06	1	-	-
17	Desmedt	Poor	GSM177901.cel.gz	14	1	-	-
18	Desmedt	Poor	GSM177902.cel.gz	56.23	1	-	-
19	Desmedt	-	GSM177903.cel.gz	92.35	1	-	-
20	Desmedt	Good	GSM177904.cel.gz	183.65	0	-	-
21	Desmedt	Good	GSM177905.cel.gz	187.84	0	-	-
22	Desmedt	Good	GSM177906.cel.gz	186.61	0	-	-
23	Desmedt	Good	GSM177907.cel.gz	182.61	0	-	-
24	Desmedt	-	GSM177908.cel.gz	93.84	1	-	-
25	Desmedt	Poor	GSM177909.cel.gz	37.77	1	-	-
26	Desmedt	Good	GSM177910.cel.gz	164.84	0	-	-
27	Desmedt	Good	GSM177911.cel.gz	178.48	0	-	-
28	Desmedt	Good	GSM177912.cel.gz	179.71	0	-	-
29	Desmedt	Good	GSM177913.cel.gz	178.03	0	-	-
30	Desmedt	Good	GSM177914.cel.gz	173.52	0	-	-
31	Desmedt	Good	GSM177915.cel.gz	150.81	0	-	-
32	Desmedt	Good	GSM177916.cel.gz	150.06	0	-	-
33	Desmedt	Good	GSM177917.cel.gz	136.35	0	-	-
34	Desmedt	Poor	GSM177918.cel.gz	29.35	1	-	-
35	Desmedt	Good	GSM177919.cel.gz	144.26	0	-	-
36	Desmedt	Poor	GSM177920.cel.gz	38.9	1	-	-
37	Desmedt	Good	GSM177921.cel.gz	227.65	0	-	-
38	Desmedt	-	GSM177922.cel.gz	30.9	0	-	-
39	Desmedt	Good	GSM177923.cel.gz	172.87	0	-	-
40	Desmedt	Good	GSM177924.cel.gz	191.77	0	-	-
41	Desmedt	Good	GSM177925.cel.gz	182.29	0	-	-
42	Desmedt	Poor	GSM177926.cel.gz	17.03	1	-	-
43	Desmedt	Poor	GSM177927.cel.gz	25.68	1	-	-
44	Desmedt	Good	GSM177928.cel.gz	183.39	0	-	-
45	Desmedt	Poor	GSM177929.cel.gz	33	1	-	-
46	Desmedt	Good	GSM177930.cel.gz	154.55	0	-	-
47	Desmedt	-	GSM177931.cel.gz	39.19	0	-	-
48	Desmedt	Good	GSM177932.cel.gz	121.9	0	-	-
49	Desmedt	Good	GSM177933.cel.gz	171.16	0	-	-
50	Desmedt	Good	GSM177934.cel.gz	183.16	0	-	-
51	Desmedt	Poor	GSM177935.cel.gz	13.58	1	-	-
52	Desmedt	Good	GSM177936.cel.gz	181.03	0	-	-
53	Desmedt	-	GSM177937.cel.gz	100.68	1	-	-
54	Desmedt	-	GSM177938.cel.gz	35.61	0	-	-
55	Desmedt	Good	GSM177939.cel.gz	151.32	0	-	-
56	Desmedt	Good	GSM177940.cel.gz	168.97	0	-	-
57	Desmedt	Good	GSM177941.cel.gz	164.55	0	-	-
58	Desmedt	Good	GSM177942.cel.gz	90.61	0	-	-
59	Desmedt	Good	GSM177943.cel.gz	106.32	0	-	-
60	Desmedt	Good	GSM177944.cel.gz	114.68	0	-	-
61	Desmedt	Good	GSM177945.cel.gz	175.26	0	-	-
62	Desmedt	Good	GSM177946.cel.gz	168.65	0	-	-
63	Desmedt	Good	GSM177947.cel.gz	171.48	0	-	-
64	Desmedt	Good	GSM177948.cel.gz	158.87	0	-	-
65	Desmedt	Good	GSM177949.cel.gz	161.68	0	-	-
66	Desmedt	Good	GSM177950.cel.gz	149.74	0	-	-
67	Desmedt	Good	GSM177951.cel.gz	106.81	0	-	-
68	Desmedt	Good	GSM177952.cel.gz	156.87	0	-	-
69	Desmedt	Poor	GSM177953.cel.gz	58.55	1	-	-
70	Desmedt	Poor	GSM177954.cel.gz	25.9	1	-	-
71	Desmedt	Good	GSM177955.cel.gz	67.9	0	-	-
72	Desmedt	Poor	GSM177956.cel.gz	17.1	1	-	-
73	Desmedt	Good	GSM177957.cel.gz	155.81	0	-	-

74	Desmedt	Poor	GSM177958.cel.gz	55.52	1	-	-
75	Desmedt	Good	GSM177959.cel.gz	152.26	0	-	-
76	Desmedt	Poor	GSM177960.cel.gz	40.61	1	-	-
77	Desmedt	-	GSM177961.cel.gz	128.26	1	-	-
78	Desmedt	-	GSM177962.cel.gz	130.65	1	-	-
79	Desmedt	-	GSM177963.cel.gz	56.13	0	-	-
80	Desmedt	-	GSM177964.cel.gz	116.39	1	-	-
81	Desmedt	Good	GSM177965.cel.gz	125.81	0	-	-
82	Desmedt	-	GSM177966.cel.gz	50.13	0	-	-
83	Desmedt	Good	GSM177967.cel.gz	114.32	0	-	-
84	Desmedt	Good	GSM177968.cel.gz	120.03	0	-	-
85	Desmedt	Good	GSM177969.cel.gz	121.65	0	-	-
86	Desmedt	Good	GSM177970.cel.gz	106.23	0	-	-
87	Desmedt	Good	GSM177971.cel.gz	122.58	0	-	-
88	Desmedt	-	GSM177972.cel.gz	142.42	1	-	-
89	Desmedt	Poor	GSM177973.cel.gz	20.94	1	-	-
90	Desmedt	Good	GSM177974.cel.gz	159.74	0	-	-
91	Desmedt	Poor	GSM177975.cel.gz	54.61	1	-	-
92	Desmedt	Good	GSM177976.cel.gz	148.48	0	-	-
93	Desmedt	Good	GSM177977.cel.gz	156.68	0	-	-
94	Desmedt	Good	GSM177978.cel.gz	151	0	-	-
95	Desmedt	-	GSM177979.cel.gz	95.65	1	-	-
96	Desmedt	Good	GSM177980.cel.gz	148.26	0	-	-
97	Desmedt	Poor	GSM177981.cel.gz	35.87	1	-	-
98	Desmedt	-	GSM177982.cel.gz	61.87	1	-	-
99	Desmedt	Poor	GSM177983.cel.gz	55.84	1	-	-
100	Desmedt	Good	GSM177984.cel.gz	139.58	0	-	-
101	Desmedt	Good	GSM177985.cel.gz	140.39	0	-	-
102	Desmedt	Good	GSM177986.cel.gz	134.87	0	-	-
103	Desmedt	Good	GSM177987.cel.gz	102.84	0	-	-
104	Desmedt	Good	GSM177988.cel.gz	86.19	0	-	-
105	Desmedt	Poor	GSM177989.cel.gz	23.55	1	-	-
106	Desmedt	-	GSM177990.cel.gz	51.32	0	-	-
107	Desmedt	Good	GSM177991.cel.gz	61.94	0	-	-
108	Desmedt	Good	GSM177992.cel.gz	85.39	0	-	-
109	Desmedt	Poor	GSM177993.cel.gz	8.68	1	-	-
110	Desmedt	-	GSM177994.cel.gz	41.61	0	-	-
111	Desmedt	Good	GSM178046.cel.gz	203.06	0	-	-
112	Desmedt	-	GSM178047.cel.gz	231.77	1	-	-
113	Desmedt	Good	GSM178048.cel.gz	202.61	0	-	-
114	Desmedt	-	GSM178049.cel.gz	106.87	1	-	-
115	Desmedt	Good	GSM178050.cel.gz	193.65	0	-	-
116	Desmedt	-	GSM178051.cel.gz	95.87	1	-	-
117	Desmedt	Good	GSM178052.cel.gz	201.03	0	-	-
118	Desmedt	Good	GSM178053.cel.gz	293.81	0	-	-
119	Desmedt	-	GSM178054.cel.gz	140.42	1	-	-
120	Desmedt	Good	GSM178055.cel.gz	126.26	0	-	-
121	Desmedt	Good	GSM178056.cel.gz	167.13	0	-	-
122	Desmedt	Good	GSM178057.cel.gz	124.35	0	-	-
123	Desmedt	Good	GSM178058.cel.gz	166.32	0	-	-
124	Desmedt	Good	GSM178059.cel.gz	153.03	0	-	-
125	Desmedt	Poor	GSM178060.cel.gz	25.61	1	-	-
126	Desmedt	Poor	GSM178061.cel.gz	51.55	1	-	-
127	Desmedt	Good	GSM178062.cel.gz	162.52	0	-	-
128	Desmedt	Good	GSM178063.cel.gz	157.23	0	-	-
129	Desmedt	Good	GSM178064.cel.gz	146.94	0	-	-
130	Desmedt	Good	GSM178065.cel.gz	144.74	0	-	-
131	Desmedt	Good	GSM178066.cel.gz	138.61	0	-	-
132	Desmedt	Good	GSM178067.cel.gz	156.97	0	-	-
133	Desmedt	Good	GSM178068.cel.gz	134.77	0	-	-
134	Desmedt	-	GSM178069.cel.gz	148.97	1	-	-
135	Desmedt	Good	GSM178070.cel.gz	130.71	0	-	-
136	Desmedt	Good	GSM178071.cel.gz	122	0	-	-
137	Desmedt	Good	GSM178072.cel.gz	112.65	0	-	-
138	Desmedt	Good	GSM178073.cel.gz	82.61	0	-	-
139	Desmedt	-	GSM178074.cel.gz	79.16	1	-	-
140	Desmedt	Good	GSM178075.cel.gz	92.87	0	-	-
141	Desmedt	Good	GSM178076.cel.gz	165.71	0	-	-
142	Desmedt	Good	GSM178077.cel.gz	226.55	0	-	-
143	Desmedt	Good	GSM178078.cel.gz	183	0	-	-
144	Desmedt	Poor	GSM178079.cel.gz	13.03	1	-	-
145	Desmedt	Good	GSM178080.cel.gz	71.77	0	-	-
146	Desmedt	Good	GSM178081.cel.gz	87.81	0	-	-
147	Desmedt	-	GSM178082.cel.gz	57.45	0	-	-

148	Miller	Good	GSM79114.CEL.gz	-	-	141.96	0
149	Miller	Good	GSM79115.CEL.gz	-	-	141.96	0
150	Miller	Good	GSM79116.CEL.gz	-	-	141.96	0
151	Miller	-	GSM79117.CEL.gz	-	-	-	-
152	Miller	-	GSM79118.CEL.gz	-	-	42.96	0
153	Miller	Good	GSM79119.CEL.gz	-	-	141	0
154	Miller	-	GSM79120.CEL.gz	-	-	83.04	1
155	Miller	Good	GSM79121.CEL.gz	-	-	135.96	0
156	Miller	Good	GSM79122.CEL.gz	-	-	140.04	0
157	Miller	-	GSM79123.CEL.gz	-	-	86.04	1
158	Miller	Poor	GSM79124.CEL.gz	-	-	18	1
159	Miller	Good	GSM79125.CEL.gz	-	-	66	0
160	Miller	Good	GSM79126.CEL.gz	-	-	134.04	0
161	Miller	Good	GSM79127.CEL.gz	-	-	89.04	0
162	Miller	Good	GSM79128.CEL.gz	-	-	138.96	0
163	Miller	Good	GSM79129.CEL.gz	-	-	134.04	0
164	Miller	-	GSM79130.CEL.gz	-	-	108	1
165	Miller	Poor	GSM79131.CEL.gz	-	-	56.04	1
166	Miller	-	GSM79132.CEL.gz	-	-	54.96	0
167	Miller	-	GSM79133.CEL.gz	-	-	66.96	1
168	Miller	-	GSM79134.CEL.gz	-	-	69.96	1
169	Miller	-	GSM79135.CEL.gz	-	-	99	1
170	Miller	Good	GSM79136.CEL.gz	-	-	138	0
171	Miller	Good	GSM79137.CEL.gz	-	-	138	0
172	Miller	Good	GSM79138.CEL.gz	-	-	138	0
173	Miller	Good	GSM79139.CEL.gz	-	-	138	0
174	Miller	Poor	GSM79140.CEL.gz	-	-	3.96	1
175	Miller	Good	GSM79141.CEL.gz	-	-	129.96	0
176	Miller	-	GSM79142.CEL.gz	-	-	-	-
177	Miller	Good	GSM79143.CEL.gz	-	-	138	0
178	Miller	-	GSM79144.CEL.gz	-	-	26.04	0
179	Miller	Good	GSM79145.CEL.gz	-	-	138	0
180	Miller	Good	GSM79146.CEL.gz	-	-	137.04	0
181	Miller	-	GSM79147.CEL.gz	-	-	-	-
182	Miller	Good	GSM79148.CEL.gz	-	-	60.96	0
183	Miller	Poor	GSM79149.CEL.gz	-	-	35.04	1
184	Miller	Good	GSM79150.CEL.gz	-	-	129.96	0
185	Miller	Good	GSM79151.CEL.gz	-	-	137.04	0
186	Miller	Good	GSM79152.CEL.gz	-	-	137.04	0
187	Miller	-	GSM79153.CEL.gz	-	-	24.96	0
188	Miller	Good	GSM79154.CEL.gz	-	-	137.04	0
189	Miller	Poor	GSM79155.CEL.gz	-	-	54.96	1
190	Miller	Good	GSM79156.CEL.gz	-	-	137.04	0
191	Miller	Good	GSM79157.CEL.gz	-	-	137.04	0
192	Miller	Good	GSM79158.CEL.gz	-	-	102	0
193	Miller	Good	GSM79159.CEL.gz	-	-	137.04	0
194	Miller	-	GSM79160.CEL.gz	-	-	53.04	0
195	Miller	Good	GSM79161.CEL.gz	-	-	66	0
196	Miller	Good	GSM79162.CEL.gz	-	-	137.04	0
197	Miller	Good	GSM79163.CEL.gz	-	-	129	0
198	Miller	Good	GSM79164.CEL.gz	-	-	135.96	0
199	Miller	Good	GSM79165.CEL.gz	-	-	135.96	0
200	Miller	Poor	GSM79166.CEL.gz	-	-	27.96	1
201	Miller	Good	GSM79167.CEL.gz	-	-	135.96	0
202	Miller	Good	GSM79168.CEL.gz	-	-	63.96	0
203	Miller	Good	GSM79169.CEL.gz	-	-	135.96	0
204	Miller	Poor	GSM79170.CEL.gz	-	-	51.96	1
205	Miller	Poor	GSM79171.CEL.gz	-	-	48.96	1
206	Miller	Good	GSM79173.CEL.gz	-	-	135	0
207	Miller	Good	GSM79174.CEL.gz	-	-	135	0
208	Miller	Good	GSM79175.CEL.gz	-	-	69	0
209	Miller	Poor	GSM79176.CEL.gz	-	-	5.04	1
210	Miller	Good	GSM79177.CEL.gz	-	-	72	0
211	Miller	Good	GSM79178.CEL.gz	-	-	134.04	0
212	Miller	Good	GSM79179.CEL.gz	-	-	89.04	0
213	Miller	Poor	GSM79180.CEL.gz	-	-	48	1
214	Miller	-	GSM79181.CEL.gz	-	-	23.04	0
215	Miller	Good	GSM79182.CEL.gz	-	-	87	0
216	Miller	Good	GSM79183.CEL.gz	-	-	132.96	0
217	Miller	Good	GSM79184.CEL.gz	-	-	132.96	0
218	Miller	-	GSM79185.CEL.gz	-	-	129.96	1
219	Miller	Good	GSM79186.CEL.gz	-	-	132	0
220	Miller	-	GSM79187.CEL.gz	-	-	-	-
221	Miller	Poor	GSM79188.CEL.gz	-	-	17.04	1

222	Miller	Poor	GSM79189.CEL.gz	-	-	0	1
223	Miller	Good	GSM79190.CEL.gz	-	-	132	0
224	Miller	-	GSM79191.CEL.gz	-	-	-	-
225	Miller	Good	GSM79192.CEL.gz	-	-	129	0
226	Miller	Good	GSM79193.CEL.gz	-	-	132	0
227	Miller	Good	GSM79195.CEL.gz	-	-	131.04	0
228	Miller	-	GSM79196.CEL.gz	-	-	42.96	0
229	Miller	-	GSM79197.CEL.gz	-	-	36	0
230	Miller	Good	GSM79198.CEL.gz	-	-	131.04	0
231	Miller	Good	GSM79199.CEL.gz	-	-	131.04	0
232	Miller	Good	GSM79200.CEL.gz	-	-	131.04	0
233	Miller	Good	GSM79201.CEL.gz	-	-	131.04	0
234	Miller	Poor	GSM79202.CEL.gz	-	-	50.04	1
235	Miller	Good	GSM79203.CEL.gz	-	-	131.04	0
236	Miller	Good	GSM79204.CEL.gz	-	-	131.04	0
237	Miller	Good	GSM79205.CEL.gz	-	-	129.96	0
238	Miller	Good	GSM79206.CEL.gz	-	-	129.96	0
239	Miller	Good	GSM79207.CEL.gz	-	-	126.96	0
240	Miller	Poor	GSM79208.CEL.gz	-	-	53.04	1
241	Miller	Good	GSM79209.CEL.gz	-	-	77.04	0
242	Miller	Good	GSM79210.CEL.gz	-	-	129.96	0
243	Miller	-	GSM79211.CEL.gz	-	-	0.96	0
244	Miller	Good	GSM79212.CEL.gz	-	-	129.96	0
245	Miller	Good	GSM79213.CEL.gz	-	-	129.96	0
246	Miller	-	GSM79214.CEL.gz	-	-	18	0
247	Miller	Good	GSM79215.CEL.gz	-	-	129.96	0
248	Miller	Good	GSM79216.CEL.gz	-	-	120.96	0
249	Miller	Good	GSM79217.CEL.gz	-	-	129	0
250	Miller	Good	GSM79218.CEL.gz	-	-	129	0
251	Miller	Good	GSM79219.CEL.gz	-	-	129	0
252	Miller	Good	GSM79220.CEL.gz	-	-	128.04	0
253	Miller	Good	GSM79221.CEL.gz	-	-	129	0
254	Miller	Good	GSM79222.CEL.gz	-	-	128.04	0
255	Miller	Poor	GSM79223.CEL.gz	-	-	14.04	1
256	Miller	-	GSM79224.CEL.gz	-	-	-	-
257	Miller	Good	GSM79225.CEL.gz	-	-	95.04	0
258	Miller	Good	GSM79226.CEL.gz	-	-	129	0
259	Miller	Good	GSM79227.CEL.gz	-	-	129	0
260	Miller	-	GSM79228.CEL.gz	-	-	-	-
261	Miller	Good	GSM79229.CEL.gz	-	-	128.04	0
262	Miller	-	GSM79230.CEL.gz	-	-	57.96	0
263	Miller	Poor	GSM79231.CEL.gz	-	-	6.96	1
264	Miller	-	GSM79232.CEL.gz	-	-	120	1
265	Miller	Poor	GSM79233.CEL.gz	-	-	35.04	1
266	Miller	Good	GSM79234.CEL.gz	-	-	128.04	0
267	Miller	Good	GSM79235.CEL.gz	-	-	128.04	0
268	Miller	Good	GSM79236.CEL.gz	-	-	128.04	0
269	Miller	Good	GSM79237.CEL.gz	-	-	128.04	0
270	Miller	Good	GSM79238.CEL.gz	-	-	128.04	0
271	Miller	Good	GSM79239.CEL.gz	-	-	128.04	0
272	Miller	Good	GSM79240.CEL.gz	-	-	126.96	0
273	Miller	Good	GSM79241.CEL.gz	-	-	128.04	0
274	Miller	Poor	GSM79242.CEL.gz	-	-	42.96	1
275	Miller	-	GSM79243.CEL.gz	-	-	81	1
276	Miller	Good	GSM79244.CEL.gz	-	-	126.96	0
277	Miller	Good	GSM79245.CEL.gz	-	-	126.96	0
278	Miller	Good	GSM79246.CEL.gz	-	-	126.96	0
279	Miller	Poor	GSM79247.CEL.gz	-	-	12.96	1
280	Miller	Good	GSM79248.CEL.gz	-	-	122.04	0
281	Miller	Good	GSM79249.CEL.gz	-	-	126	0
282	Miller	Good	GSM79250.CEL.gz	-	-	126	0
283	Miller	-	GSM79251.CEL.gz	-	-	2.04	0
284	Miller	Good	GSM79252.CEL.gz	-	-	128.04	0
285	Miller	-	GSM79253.CEL.gz	-	-	32.04	0
286	Miller	Good	GSM79254.CEL.gz	-	-	126	0
287	Miller	Poor	GSM79255.CEL.gz	-	-	42.96	1
288	Miller	Good	GSM79256.CEL.gz	-	-	96	0
289	Miller	Good	GSM79257.CEL.gz	-	-	126	0
290	Miller	Good	GSM79258.CEL.gz	-	-	126	0
291	Miller	Poor	GSM79259.CEL.gz	-	-	33	1
292	Miller	Good	GSM79260.CEL.gz	-	-	126	0
293	Miller	Good	GSM79261.CEL.gz	-	-	123.96	0
294	Miller	Good	GSM79262.CEL.gz	-	-	125.04	0
295	Miller	Good	GSM79263.CEL.gz	-	-	125.04	0

296	Miller	Good	GSM79264.CEL.gz	-	-	125.04	0
297	Miller	-	GSM79265.CEL.gz	-	-	-	-
298	Miller	-	GSM79266.CEL.gz	-	-	99	1
299	Miller	Good	GSM79267.CEL.gz	-	-	125.04	0
300	Miller	Good	GSM79268.CEL.gz	-	-	125.04	0
301	Miller	Good	GSM79269.CEL.gz	-	-	123.96	0
302	Miller	Good	GSM79270.CEL.gz	-	-	123.96	0
303	Miller	-	GSM79271.CEL.gz	-	-	0	0
304	Miller	Poor	GSM79272.CEL.gz	-	-	17.04	1
305	Miller	Good	GSM79273.CEL.gz	-	-	123.96	0
306	Miller	Poor	GSM79274.CEL.gz	-	-	21.96	1
307	Miller	Good	GSM79275.CEL.gz	-	-	123.96	0
308	Miller	Good	GSM79276.CEL.gz	-	-	123.96	0
309	Miller	Good	GSM79277.CEL.gz	-	-	123	0
310	Miller	Good	GSM79278.CEL.gz	-	-	102.96	0
311	Miller	Good	GSM79279.CEL.gz	-	-	123	0
312	Miller	Good	GSM79280.CEL.gz	-	-	122.04	0
313	Miller	Good	GSM79281.CEL.gz	-	-	81.96	0
314	Miller	Good	GSM79282.CEL.gz	-	-	120.96	0
315	Miller	-	GSM79283.CEL.gz	-	-	-	-
316	Miller	-	GSM79284.CEL.gz	-	-	81.96	1
317	Miller	Good	GSM79285.CEL.gz	-	-	120.96	0
318	Miller	Good	GSM79286.CEL.gz	-	-	120	0
319	Miller	Good	GSM79287.CEL.gz	-	-	120	0
320	Miller	Good	GSM79288.CEL.gz	-	-	120	0
321	Miller	Good	GSM79289.CEL.gz	-	-	120	0
322	Miller	Good	GSM79290.CEL.gz	-	-	126	0
323	Miller	Good	GSM79291.CEL.gz	-	-	120	0
324	Miller	-	GSM79292.CEL.gz	-	-	11.04	0
325	Miller	Good	GSM79293.CEL.gz	-	-	120	0
326	Miller	Good	GSM79294.CEL.gz	-	-	120	0
327	Miller	Good	GSM79295.CEL.gz	-	-	119.04	0
328	Miller	Good	GSM79296.CEL.gz	-	-	119.04	0
329	Miller	Good	GSM79297.CEL.gz	-	-	119.04	0
330	Miller	Good	GSM79298.CEL.gz	-	-	119.04	0
331	Miller	Good	GSM79299.CEL.gz	-	-	119.04	0
332	Miller	Poor	GSM79300.CEL.gz	-	-	35.04	1
333	Miller	Good	GSM79301.CEL.gz	-	-	117.96	0
334	Miller	-	GSM79302.CEL.gz	-	-	63	1
335	Miller	Good	GSM79303.CEL.gz	-	-	117.96	0
336	Miller	Poor	GSM79304.CEL.gz	-	-	21	1
337	Miller	Good	GSM79305.CEL.gz	-	-	122.04	0
338	Miller	-	GSM79306.CEL.gz	-	-	114.96	1
339	Miller	Good	GSM79307.CEL.gz	-	-	96	0
340	Miller	Good	GSM79308.CEL.gz	-	-	129	0
341	Miller	-	GSM79309.CEL.gz	-	-	-	-
342	Miller	-	GSM79310.CEL.gz	-	-	-	-
343	Miller	Good	GSM79311.CEL.gz	-	-	72.96	0
344	Miller	Good	GSM79312.CEL.gz	-	-	120.96	0
345	Miller	Good	GSM79313.CEL.gz	-	-	120	0
346	Miller	Good	GSM79315.CEL.gz	-	-	120	0
347	Miller	Good	GSM79316.CEL.gz	-	-	119.04	0
348	Miller	Good	GSM79317.CEL.gz	-	-	119.04	0
349	Miller	-	GSM79318.CEL.gz	-	-	-	-
350	Miller	Good	GSM79319.CEL.gz	-	-	153	0
351	Miller	Good	GSM79320.CEL.gz	-	-	153	0
352	Miller	Poor	GSM79321.CEL.gz	-	-	0.96	1
353	Miller	Good	GSM79322.CEL.gz	-	-	126.96	0
354	Miller	-	GSM79323.CEL.gz	-	-	21.96	0
355	Miller	-	GSM79324.CEL.gz	-	-	80.04	1
356	Miller	-	GSM79325.CEL.gz	-	-	114.96	1
357	Miller	-	GSM79326.CEL.gz	-	-	-	-
358	Miller	Good	GSM79327.CEL.gz	-	-	152.04	0
359	Miller	Poor	GSM79328.CEL.gz	-	-	38.04	1
360	Miller	Good	GSM79329.CEL.gz	-	-	150.96	0
361	Miller	-	GSM79330.CEL.gz	-	-	39	0
362	Miller	Poor	GSM79331.CEL.gz	-	-	27	1
363	Miller	Poor	GSM79332.CEL.gz	-	-	15.96	1
364	Miller	-	GSM79333.CEL.gz	-	-	66	1
365	Miller	Poor	GSM79334.CEL.gz	-	-	11.04	1
366	Miller	Good	GSM79335.CEL.gz	-	-	150	0
367	Miller	Poor	GSM79336.CEL.gz	-	-	3.96	1
368	Miller	Poor	GSM79337.CEL.gz	-	-	60	1
369	Miller	Good	GSM79338.CEL.gz	-	-	149.04	0

370	Miller	Good	GSM79339.CEL.gz	-	-	149.04	0
371	Miller	Poor	GSM79340.CEL.gz	-	-	17.04	1
372	Miller	Poor	GSM79341.CEL.gz	-	-	14.04	1
373	Miller	Good	GSM79342.CEL.gz	-	-	149.04	0
374	Miller	Poor	GSM79343.CEL.gz	-	-	20.04	1
375	Miller	Good	GSM79344.CEL.gz	-	-	144	0
376	Miller	Good	GSM79345.CEL.gz	-	-	147.96	0
377	Miller	-	GSM79346.CEL.gz	-	-	-	-
378	Miller	Good	GSM79347.CEL.gz	-	-	147.96	0
379	Miller	-	GSM79348.CEL.gz	-	-	143.04	1
380	Miller	-	GSM79349.CEL.gz	-	-	24.96	0
381	Miller	-	GSM79351.CEL.gz	-	-	9.96	0
382	Miller	Poor	GSM79352.CEL.gz	-	-	54.96	1
383	Miller	-	GSM79353.CEL.gz	-	-	30.96	0
384	Miller	Good	GSM79354.CEL.gz	-	-	146.04	0
385	Miller	-	GSM79355.CEL.gz	-	-	24.96	0
386	Miller	Good	GSM79356.CEL.gz	-	-	146.04	0
387	Miller	Good	GSM79357.CEL.gz	-	-	144.96	0
388	Miller	Good	GSM79358.CEL.gz	-	-	144.96	0
389	Miller	Good	GSM79359.CEL.gz	-	-	135.96	0
390	Miller	-	GSM79360.CEL.gz	-	-	32.04	0
391	Miller	-	GSM79361.CEL.gz	-	-	-	-
392	Miller	Poor	GSM79362.CEL.gz	-	-	3	1
393	Miller	Good	GSM79363.CEL.gz	-	-	126	0
394	Miller	Good	GSM79364.CEL.gz	-	-	135.96	0
395	Minn	Good	GSM50034.CEL.GZ	86.96	0	-	-
396	Minn	Poor	GSM50035.CEL.GZ	22.03	1	-	-
397	Minn	Good	GSM50036.CEL.GZ	109.74	0	-	-
398	Minn	Good	GSM50037.CEL.GZ	82.29	0	-	-
399	Minn	Good	GSM50038.CEL.GZ	77.62	0	-	-
400	Minn	Good	GSM50039.CEL.GZ	85.02	0	-	-
401	Minn	Good	GSM50040.CEL.GZ	90.84	0	-	-
402	Minn	-	GSM50041.CEL.GZ	0	-	-	-
403	Minn	-	GSM50042.CEL.GZ	52.93	0	-	-
404	Minn	Good	GSM50043.CEL.GZ	62.79	0	-	-
405	Minn	Good	GSM50044.CEL.GZ	67.46	0	-	-
406	Minn	Good	GSM50045.CEL.GZ	129	0	-	-
407	Minn	Good	GSM50046.CEL.GZ	96.46	0	-	-
408	Minn	Good	GSM50047.CEL.GZ	64.87	0	-	-
409	Minn	Good	GSM50048.CEL.GZ	72.85	0	-	-
410	Minn	Good	GSM50049.CEL.GZ	65.95	0	-	-
411	Minn	-	GSM50050.CEL.GZ	0	-	-	-
412	Minn	-	GSM50051.CEL.GZ	45.21	0	-	-
413	Minn	-	GSM50052.CEL.GZ	54.77	0	-	-
414	Minn	-	GSM50053.CEL.GZ	47.61	0	-	-
415	Minn	-	GSM50054.CEL.GZ	48.59	0	-	-
416	Minn	-	GSM50055.CEL.GZ	0	-	-	-
417	Minn	-	GSM50056.CEL.GZ	0	-	-	-
418	Minn	-	GSM50057.CEL.GZ	0	-	-	-
419	Minn	-	GSM50058.CEL.GZ	0	-	-	-
420	Minn	Good	GSM50059.CEL.GZ	91.59	0	-	-
421	Minn	Poor	GSM50060.CEL.GZ	41.69	1	-	-
422	Minn	Poor	GSM50061.CEL.GZ	47.51	1	-	-
423	Minn	Poor	GSM50062.CEL.GZ	36.79	1	-	-
424	Minn	Poor	GSM50063.CEL.GZ	37.35	1	-	-
425	Minn	Poor	GSM50064.CEL.GZ	11.24	1	-	-
426	Minn	Good	GSM50065.CEL.GZ	69.73	0	-	-
427	Minn	Poor	GSM50066.CEL.GZ	14.86	1	-	-
428	Minn	Poor	GSM50067.CEL.GZ	9.27	1	-	-
429	Minn	Poor	GSM50068.CEL.GZ	41.72	1	-	-
430	Minn	Poor	GSM50069.CEL.GZ	45.93	1	-	-
431	Minn	Poor	GSM50070.CEL.GZ	40.37	1	-	-
432	Minn	Good	GSM50071.CEL.GZ	69.86	0	-	-
433	Minn	Good	GSM50072.CEL.GZ	128.84	0	-	-
434	Minn	Good	GSM50073.CEL.GZ	86.96	0	-	-
435	Minn	Good	GSM50074.CEL.GZ	87.81	0	-	-
436	Minn	Good	GSM50075.CEL.GZ	87.81	0	-	-
437	Minn	-	GSM50076.CEL.GZ	0	-	-	-
438	Minn	-	GSM50077.CEL.GZ	0	-	-	-
439	Minn	Good	GSM50078.CEL.GZ	83.8	0	-	-
440	Minn	Good	GSM50079.CEL.GZ	78.25	0	-	-
441	Minn	Good	GSM50080.CEL.GZ	86.99	0	-	-
442	Minn	Good	GSM50081.CEL.GZ	60.82	0	-	-
443	Minn	Good	GSM50082.CEL.GZ	85.32	0	-	-

444	Minn	Good	GSM50083.CEL.GZ	87.32	0	-	-
445	Minn	Good	GSM50084.CEL.GZ	76.77	0	-	-
446	Minn	Good	GSM50085.CEL.GZ	78.05	0	-	-
447	Minn	Good	GSM50086.CEL.GZ	99.75	0	-	-
448	Minn	Good	GSM50087.CEL.GZ	99.75	0	-	-
449	Minn	-	GSM50088.CEL.GZ	0	-	-	-
450	Minn	Good	GSM50089.CEL.GZ	104.91	0	-	-
451	Minn	Good	GSM50090.CEL.GZ	81.44	0	-	-
452	Minn	Good	GSM50091.CEL.GZ	86.33	0	-	-
453	Minn	Good	GSM50092.CEL.GZ	77.39	0	-	-
454	Minn	Good	GSM50093.CEL.GZ	94.29	0	-	-
455	Minn	Poor	GSM50094.CEL.GZ	38.7	1	-	-
456	Minn	-	GSM50095.CEL.GZ	78.48	1	-	-
457	Minn	Poor	GSM50096.CEL.GZ	16.6	1	-	-
458	Minn	-	GSM50097.CEL.GZ	64.73	1	-	-
459	Minn	Poor	GSM50098.CEL.GZ	30.87	1	-	-
460	Minn	Poor	GSM50099.CEL.GZ	15.62	1	-	-
461	Minn	-	GSM50100.CEL.GZ	69.7	1	-	-
462	Minn	-	GSM50101.CEL.GZ	76.37	1	-	-
463	Minn	Poor	GSM50102.CEL.GZ	7.99	1	-	-
464	Minn	Poor	GSM50103.CEL.GZ	22.03	1	-	-
465	Minn	-	GSM50104.CEL.GZ	81.27	1	-	-
466	Minn	Good	GSM50105.CEL.GZ	86.27	0	-	-
467	Minn	Poor	GSM50106.CEL.GZ	18.9	1	-	-
468	Minn	Good	GSM50107.CEL.GZ	74.56	0	-	-
469	Minn	-	GSM50109.CEL.GZ	0	-	-	-
470	Minn	Good	GSM50110.CEL.GZ	62.24	0	-	-
471	Minn	Good	GSM50111.CEL.GZ	87.65	0	-	-
472	Minn	Poor	GSM50112.CEL.GZ	14.2	1	-	-
473	Minn	-	GSM50113.CEL.GZ	0	-	-	-
474	Minn	-	GSM50115.CEL.GZ	53.06	0	-	-
475	Minn	-	GSM50116.CEL.GZ	43	0	-	-
476	Minn	-	GSM50117.CEL.GZ	0	-	-	-
477	Minn	Good	GSM50118.CEL.GZ	75.32	0	-	-
478	Minn	-	GSM50119.CEL.GZ	50.1	0	-	-
479	Minn	-	GSM50120.CEL.GZ	44.22	0	-	-
480	Minn	-	GSM50121.CEL.GZ	49.48	0	-	-
481	Minn	Poor	GSM50122.CEL.GZ	38.93	1	-	-
482	Minn	-	GSM50123.CEL.GZ	46.82	0	-	-
483	Minn	-	GSM50124.CEL.GZ	0	-	-	-
484	Minn	-	GSM50125.CEL.GZ	0	-	-	-
485	Minn	-	GSM50126.CEL.GZ	0	-	-	-
486	Minn	Good	GSM50127.CEL.GZ	62.89	0	-	-
487	Minn	-	GSM50128.CEL.GZ	27.6	0	-	-
488	Minn	-	GSM50129.CEL.GZ	0	-	-	-
489	Minn	-	GSM50130.CEL.GZ	40.31	0	-	-
490	Minn	Poor	GSM50131.CEL.GZ	55.13	1	-	-
491	Pawitan	Poor	GSM107072.CEL.GZ	-	-	49.68	1
492	Pawitan	Good	GSM107073.CEL.GZ	-	-	97.8	0
493	Pawitan	-	GSM107074.CEL.GZ	-	-	26.64	0
494	Pawitan	Good	GSM107075.CEL.GZ	-	-	98.76	0
495	Pawitan	Good	GSM107076.CEL.GZ	-	-	66.6	0
496	Pawitan	Good	GSM107077.CEL.GZ	-	-	99.6	0
497	Pawitan	-	GSM107078.CEL.GZ	-	-	53.04	0
498	Pawitan	Good	GSM107079.CEL.GZ	-	-	96.84	0
499	Pawitan	Good	GSM107080.CEL.GZ	-	-	76.56	0
500	Pawitan	Good	GSM107081.CEL.GZ	-	-	97.56	0
501	Pawitan	-	GSM107082.CEL.GZ	-	-	66.12	1
502	Pawitan	Good	GSM107083.CEL.GZ	-	-	97.32	0
503	Pawitan	-	GSM107084.CEL.GZ	-	-	65.64	1
504	Pawitan	Good	GSM107085.CEL.GZ	-	-	81.84	0
505	Pawitan	-	GSM107086.CEL.GZ	-	-	75	1
506	Pawitan	Good	GSM107087.CEL.GZ	-	-	92.52	0
507	Pawitan	-	GSM107088.CEL.GZ	-	-	74.64	1
508	Pawitan	Poor	GSM107089.CEL.GZ	-	-	36.72	1
509	Pawitan	Good	GSM107090.CEL.GZ	-	-	70.44	0
510	Pawitan	Good	GSM107091.CEL.GZ	-	-	94.32	0
511	Pawitan	Poor	GSM107092.CEL.GZ	-	-	46.2	1
512	Pawitan	Poor	GSM107093.CEL.GZ	-	-	31.8	1
513	Pawitan	-	GSM107094.CEL.GZ	-	-	67.56	1
514	Pawitan	Good	GSM107095.CEL.GZ	-	-	91.32	0
515	Pawitan	Good	GSM107096.CEL.GZ	-	-	90.6	0
516	Pawitan	Poor	GSM107097.CEL.GZ	-	-	38.16	1
517	Pawitan	Good	GSM107098.CEL.GZ	-	-	96.36	0

518	Pawitan	Good	GSM107099.CEL.GZ	-	-	87.72	0
519	Pawitan	Good	GSM107100.CEL.GZ	-	-	91.8	0
520	Pawitan	Good	GSM107101.CEL.GZ	-	-	88.56	0
521	Pawitan	Good	GSM107102.CEL.GZ	-	-	87.48	0
522	Pawitan	Good	GSM107103.CEL.GZ	-	-	96.36	0
523	Pawitan	Good	GSM107104.CEL.GZ	-	-	96.36	0
524	Pawitan	Good	GSM107105.CEL.GZ	-	-	95.16	0
525	Pawitan	Good	GSM107106.CEL.GZ	-	-	92.76	0
526	Pawitan	Good	GSM107107.CEL.GZ	-	-	98.4	0
527	Pawitan	Good	GSM107108.CEL.GZ	-	-	91.08	0
528	Pawitan	Good	GSM107109.CEL.GZ	-	-	93.72	0
529	Pawitan	Good	GSM107110.CEL.GZ	-	-	101.76	0
530	Pawitan	Good	GSM107111.CEL.GZ	-	-	95.4	0
531	Pawitan	-	GSM107112.CEL.GZ	-	-	26.88	0
532	Pawitan	Good	GSM107113.CEL.GZ	-	-	87.36	0
533	Pawitan	Good	GSM107114.CEL.GZ	-	-	95.52	0
534	Pawitan	Good	GSM107115.CEL.GZ	-	-	82.32	0
535	Pawitan	Good	GSM107117.CEL.GZ	-	-	84.36	0
536	Pawitan	Good	GSM107118.CEL.GZ	-	-	101.52	0
537	Pawitan	Good	GSM107119.CEL.GZ	-	-	94.08	0
538	Pawitan	Good	GSM107120.CEL.GZ	-	-	73.56	0
539	Pawitan	Good	GSM107121.CEL.GZ	-	-	86.52	0
540	Pawitan	Good	GSM107122.CEL.GZ	-	-	97.56	0
541	Pawitan	Good	GSM107123.CEL.GZ	-	-	91.8	0
542	Pawitan	Good	GSM107124.CEL.GZ	-	-	84.84	0
543	Pawitan	Poor	GSM107125.CEL.GZ	-	-	56.64	1
544	Pawitan	Good	GSM107126.CEL.GZ	-	-	90.6	0
545	Pawitan	Good	GSM107127.CEL.GZ	-	-	93	0
546	Pawitan	Good	GSM107128.CEL.GZ	-	-	96.96	0
547	Pawitan	Poor	GSM107129.CEL.GZ	-	-	28.56	1
548	Pawitan	Good	GSM107130.CEL.GZ	-	-	100.8	0
549	Pawitan	Good	GSM107131.CEL.GZ	-	-	90.48	0
550	Pawitan	Good	GSM107132.CEL.GZ	-	-	87.36	0
551	Pawitan	Good	GSM107133.CEL.GZ	-	-	92.76	0
552	Pawitan	Poor	GSM107134.CEL.GZ	-	-	36	1
553	Pawitan	Good	GSM107135.CEL.GZ	-	-	75.72	0
554	Pawitan	Good	GSM107136.CEL.GZ	-	-	99.6	0
555	Pawitan	Good	GSM107137.CEL.GZ	-	-	84.84	0
556	Pawitan	-	GSM107138.CEL.GZ	-	-	76.8	1
557	Pawitan	Good	GSM107139.CEL.GZ	-	-	97.8	0
558	Pawitan	Good	GSM107141.CEL.GZ	-	-	72.24	0
559	Pawitan	Good	GSM107142.CEL.GZ	-	-	70.2	0
560	Pawitan	Good	GSM107143.CEL.GZ	-	-	71.52	0
561	Pawitan	Good	GSM107144.CEL.GZ	-	-	69.72	0
562	Pawitan	Good	GSM107145.CEL.GZ	-	-	70.32	0
563	Pawitan	Good	GSM107146.CEL.GZ	-	-	69	0
564	Pawitan	Good	GSM107147.CEL.GZ	-	-	68.52	0
565	Pawitan	Good	GSM107148.CEL.GZ	-	-	67.56	0
566	Pawitan	Good	GSM107149.CEL.GZ	-	-	68.16	0
567	Pawitan	Good	GSM107150.CEL.GZ	-	-	95.88	0
568	Pawitan	Poor	GSM107152.CEL.GZ	-	-	55.32	1
569	Pawitan	Good	GSM107153.CEL.GZ	-	-	68.28	0
570	Pawitan	Good	GSM107154.CEL.GZ	-	-	69.48	0
571	Pawitan	Poor	GSM107155.CEL.GZ	-	-	20.76	1
572	Pawitan	Good	GSM107156.CEL.GZ	-	-	99.96	0
573	Pawitan	Good	GSM107157.CEL.GZ	-	-	72.36	0
574	Pawitan	-	GSM107158.CEL.GZ	-	-	54.12	0
575	Pawitan	Good	GSM107160.CEL.GZ	-	-	66.72	0
576	Pawitan	Good	GSM107161.CEL.GZ	-	-	95.04	0
577	Pawitan	Good	GSM107162.CEL.GZ	-	-	78.24	0
578	Pawitan	Good	GSM107163.CEL.GZ	-	-	89.04	0
579	Pawitan	Good	GSM107164.CEL.GZ	-	-	77.52	0
580	Pawitan	Good	GSM107165.CEL.GZ	-	-	101.16	0
581	Pawitan	-	GSM107166.CEL.GZ	-	-	80.64	1
582	Pawitan	Good	GSM107167.CEL.GZ	-	-	87.36	0
583	Pawitan	Good	GSM107168.CEL.GZ	-	-	95.52	0
584	Pawitan	Good	GSM107169.CEL.GZ	-	-	91.08	0
585	Pawitan	Poor	GSM107170.CEL.GZ	-	-	14.52	1
586	Pawitan	Good	GSM107171.CEL.GZ	-	-	90.12	0
587	Pawitan	Good	GSM107172.CEL.GZ	-	-	92.04	0
588	Pawitan	Good	GSM107173.CEL.GZ	-	-	99.6	0
589	Pawitan	Good	GSM107174.CEL.GZ	-	-	93.36	0
590	Pawitan	Good	GSM107175.CEL.GZ	-	-	94.68	0
591	Pawitan	Good	GSM107176.CEL.GZ	-	-	89.28	0

592	Pawitan	Good	GSM107177.CEL.GZ	-	-	71.4	0
593	Pawitan	Poor	GSM107178.CEL.GZ	-	-	33.96	1
594	Pawitan	Good	GSM107179.CEL.GZ	-	-	75.12	0
595	Pawitan	Good	GSM107180.CEL.GZ	-	-	99.84	0
596	Pawitan	Poor	GSM107181.CEL.GZ	-	-	57.96	1
597	Pawitan	Good	GSM107182.CEL.GZ	-	-	74.04	0
598	Pawitan	Good	GSM107183.CEL.GZ	-	-	97.56	0
599	Pawitan	Good	GSM107184.CEL.GZ	-	-	71.4	0
600	Pawitan	Good	GSM107185.CEL.GZ	-	-	71.76	0
601	Pawitan	Good	GSM107186.CEL.GZ	-	-	72.6	0
602	Pawitan	Good	GSM107187.CEL.GZ	-	-	81.12	0
603	Pawitan	Good	GSM107188.CEL.GZ	-	-	99.84	0
604	Pawitan	Good	GSM107189.CEL.GZ	-	-	78.6	0
605	Pawitan	Good	GSM107190.CEL.GZ	-	-	95.28	0
606	Pawitan	Good	GSM107191.CEL.GZ	-	-	78.6	0
607	Pawitan	Good	GSM107192.CEL.GZ	-	-	84.6	0
608	Pawitan	Good	GSM107193.CEL.GZ	-	-	76.8	0
609	Pawitan	Good	GSM107194.CEL.GZ	-	-	88.8	0
610	Pawitan	Poor	GSM107195.CEL.GZ	-	-	20.64	1
611	Pawitan	Good	GSM107196.CEL.GZ	-	-	92.52	0
612	Pawitan	Good	GSM107197.CEL.GZ	-	-	68.28	0
613	Pawitan	Good	GSM107198.CEL.GZ	-	-	92.04	0
614	Pawitan	Poor	GSM107199.CEL.GZ	-	-	57.48	1
615	Pawitan	Good	GSM107200.CEL.GZ	-	-	93.36	0
616	Pawitan	Good	GSM107201.CEL.GZ	-	-	90	0
617	Pawitan	Good	GSM107202.CEL.GZ	-	-	88.32	0
618	Pawitan	Good	GSM107203.CEL.GZ	-	-	92.76	0
619	Pawitan	Poor	GSM107204.CEL.GZ	-	-	13.68	1
620	Pawitan	Poor	GSM107205.CEL.GZ	-	-	20.04	1
621	Pawitan	Poor	GSM107206.CEL.GZ	-	-	46.08	1
622	Pawitan	Good	GSM107207.CEL.GZ	-	-	101.64	0
623	Pawitan	Good	GSM107208.CEL.GZ	-	-	96.36	0
624	Pawitan	Good	GSM107209.CEL.GZ	-	-	95.88	0
625	Pawitan	Poor	GSM107210.CEL.GZ	-	-	33.6	1
626	Pawitan	Good	GSM107211.CEL.GZ	-	-	91.56	0
627	Pawitan	Good	GSM107212.CEL.GZ	-	-	94.08	0
628	Pawitan	Good	GSM107213.CEL.GZ	-	-	93	0
629	Pawitan	Good	GSM107214.CEL.GZ	-	-	92.04	0
630	Pawitan	-	GSM107215.CEL.GZ	-	-	27.36	0
631	Pawitan	Good	GSM107216.CEL.GZ	-	-	79.32	0
632	Pawitan	Good	GSM107217.CEL.GZ	-	-	86.28	0
633	Pawitan	Good	GSM107218.CEL.GZ	-	-	77.28	0
634	Pawitan	Poor	GSM107219.CEL.GZ	-	-	52.92	1
635	Pawitan	Good	GSM107220.CEL.GZ	-	-	75.84	0
636	Pawitan	Good	GSM107221.CEL.GZ	-	-	100.32	0
637	Pawitan	Good	GSM107222.CEL.GZ	-	-	101.88	0
638	Pawitan	-	GSM107223.CEL.GZ	-	-	2.16	0
639	Pawitan	Good	GSM107224.CEL.GZ	-	-	71.28	0
640	Pawitan	Good	GSM107225.CEL.GZ	-	-	99.84	0
641	Pawitan	Good	GSM107226.CEL.GZ	-	-	71.76	0
642	Pawitan	Good	GSM107227.CEL.GZ	-	-	72.24	0
643	Pawitan	Poor	GSM107228.CEL.GZ	-	-	36.84	1
644	Pawitan	Poor	GSM107229.CEL.GZ	-	-	58.44	1
645	Pawitan	Good	GSM107230.CEL.GZ	-	-	67.44	0
646	Pawitan	-	GSM107231.CEL.GZ	-	-	8.4	0
647	Loi	-	GSM150794.CEL.gz	153.94	-	-	-
648	Loi	-	GSM150795.CEL.gz	24.81	-	-	-
649	Loi	-	GSM150796.CEL.gz	127.06	-	-	-
650	Loi	-	GSM150797.CEL.gz	7.55	-	-	-
651	Loi	-	GSM150798.CEL.gz	44.19	-	-	-
652	Loi	-	GSM150799.CEL.gz	146.81	-	-	-
653	Loi	-	GSM150800.CEL.gz	85.52	-	-	-
654	Loi	-	GSM150801.CEL.gz	123.23	-	-	-
655	Loi	Good	gsm65820.cel.gz	171.13	0	-	-
656	Loi	Good	gsm65821.cel.gz	131.65	0	-	-
657	Loi	Good	gsm65822.cel.gz	99.81	0	-	-
658	Loi	Good	gsm65823.cel.gz	167.03	0	-	-
659	Loi	-	gsm65824.cel.gz	141.87	1	-	-
660	Loi	Good	gsm65825.cel.gz	164.39	0	-	-
661	Loi	-	gsm65826.cel.gz	68.55	1	-	-
662	Loi	Good	gsm65827.cel.gz	162.23	0	-	-
663	Loi	Poor	gsm65828.cel.gz	20.9	1	-	-
664	Loi	Good	gsm65829.cel.gz	134.29	0	-	-
665	Loi	Good	gsm65830.cel.gz	157.06	0	-	-

666	Loi	Good	gsm65831.cel.gz	162.23	0	-	-
667	Loi	Good	gsm65832.cel.gz	152.06	0	-	-
668	Loi	Good	gsm65833.cel.gz	158.26	0	-	-
669	Loi	-	gsm65834.cel.gz	147.71	1	-	-
670	Loi	Poor	gsm65835.cel.gz	18.13	1	-	-
671	Loi	Good	gsm65836.cel.gz	148.55	0	-	-
672	Loi	-	gsm65837.cel.gz	60.48	1	-	-
673	Loi	Good	gsm65838.cel.gz	156.74	0	-	-
674	Loi	-	gsm65839.cel.gz	18.13	0	-	-
675	Loi	Good	gsm65840.cel.gz	148.84	0	-	-
676	Loi	Good	gsm65841.cel.gz	124.32	0	-	-
677	Loi	Good	gsm65842.cel.gz	151.06	0	-	-
678	Loi	Good	gsm65843.cel.gz	82.65	0	-	-
679	Loi	Poor	gsm65844.cel.gz	7.13	1	-	-
680	Loi	Good	gsm65845.cel.gz	148.32	0	-	-
681	Loi	Poor	gsm65846.cel.gz	35.87	1	-	-
682	Loi	Poor	gsm65847.cel.gz	34.35	1	-	-
683	Loi	Good	gsm65848.cel.gz	150.58	0	-	-
684	Loi	Poor	gsm65849.cel.gz	34.06	1	-	-
685	Loi	Good	gsm65850.cel.gz	146.26	0	-	-
686	Loi	Good	gsm65851.cel.gz	146.77	0	-	-
687	Loi	Poor	gsm65852.cel.gz	40.87	1	-	-
688	Loi	Good	gsm65853.cel.gz	126.19	0	-	-
689	Loi	Good	gsm65854.cel.gz	144.42	0	-	-
690	Loi	-	gsm65855.cel.gz	36.65	0	-	-
691	Loi	Good	gsm65856.cel.gz	115.58	0	-	-
692	Loi	Poor	gsm65857.cel.gz	55.84	1	-	-
693	Loi	Good	gsm65858.cel.gz	141.77	0	-	-
694	Loi	Good	gsm65859.cel.gz	145.1	0	-	-
695	Loi	Good	gsm65860.cel.gz	144.29	0	-	-
696	Loi	Poor	gsm65861.cel.gz	31.13	1	-	-
697	Loi	Good	gsm65862.cel.gz	139.65	0	-	-
698	Loi	Good	gsm65863.cel.gz	140.45	0	-	-
699	Loi	Good	gsm65864.cel.gz	118.26	0	-	-
700	Loi	Good	gsm65865.cel.gz	142	0	-	-
701	Loi	Good	gsm65866.cel.gz	131.55	0	-	-
702	Loi	Good	gsm65867.cel.gz	136.06	0	-	-
703	Loi	Poor	gsm65868.cel.gz	35.42	1	-	-
704	Loi	Poor	gsm65869.cel.gz	31	1	-	-
705	Loi	Good	gsm65870.cel.gz	134.94	0	-	-
706	Loi	Good	gsm65871.cel.gz	102.87	0	-	-
707	Loi	Poor	gsm65872.cel.gz	23.52	1	-	-
708	Loi	-	gsm65873.cel.gz	51.32	0	-	-
709	Loi	Good	gsm65874.cel.gz	61.94	0	-	-
710	Loi	Good	gsm65875.cel.gz	105.23	0	-	-
711	Loi	Good	gsm65876.cel.gz	61.71	0	-	-
712	Loi	Good	gsm65877.cel.gz	85.42	0	-	-
713	Loi	Poor	gsm65878.cel.gz	8.65	1	-	-
714	Loi	-	gsm65879.cel.gz	41.61	0	-	-
715	Loi	Good	gsm65880.cel.gz	90.61	0	-	-
716	Loi	-	GSM150943.CEL.gz	31.52	0	-	-
717	Loi	Good	GSM150944.CEL.gz	91.13	0	-	-
718	Loi	-	GSM150945.CEL.gz	73.1	1	-	-
719	Loi	Poor	GSM150946.CEL.gz	43.77	1	-	-
720	Loi	Poor	GSM150947.CEL.gz	26.94	1	-	-
721	Loi	Good	GSM150948.CEL.gz	85.74	0	-	-
722	Loi	Good	GSM150949.CEL.gz	91.77	0	-	-
723	Loi	Good	GSM150950.CEL.gz	60.16	0	-	-
724	Loi	Good	GSM150951.CEL.gz	60.74	0	-	-
725	Loi	-	GSM150952.CEL.gz	52.9	0	-	-
726	Loi	Good	GSM150953.CEL.gz	94.19	0	-	-
727	Loi	Good	GSM150954.CEL.gz	73.61	0	-	-
728	Loi	-	GSM150955.CEL.gz	48.52	0	-	-
729	Loi	Poor	GSM150956.CEL.gz	36.55	1	-	-
730	Loi	Good	GSM150957.CEL.gz	81.58	0	-	-
731	Loi	Good	GSM150958.CEL.gz	84.65	0	-	-
732	Loi	Good	GSM150959.CEL.gz	82.87	0	-	-
733	Loi	Poor	GSM150960.CEL.gz	23.26	1	-	-
734	Loi	Poor	GSM150961.CEL.gz	23.13	1	-	-
735	Loi	-	GSM150962.CEL.gz	47.48	0	-	-
736	Loi	Good	GSM150963.CEL.gz	93.23	0	-	-
737	Loi	-	GSM150964.CEL.gz	47.03	0	-	-
738	Loi	Good	GSM150965.CEL.gz	80.74	0	-	-
739	Loi	Good	GSM150966.CEL.gz	70.26	0	-	-

740	Loi	-	GSM150967.CEL.gz	0.48	-	-	-
741	Loi	-	GSM150968.CEL.gz	33.65	0	-	-
742	Loi	Poor	GSM150969.CEL.gz	42.74	1	-	-
743	Loi	-	GSM150970.CEL.gz	56.74	0	-	-
744	Loi	-	GSM150971.CEL.gz	58.13	0	-	-
745	Loi	-	GSM150972.CEL.gz	58.81	0	-	-
746	Loi	Good	GSM150973.CEL.gz	63.06	0	-	-
747	Loi	Poor	GSM150974.CEL.gz	44.52	1	-	-
748	Loi	-	GSM150975.CEL.gz	38.94	0	-	-
749	Loi	-	GSM150976.CEL.gz	58.32	0	-	-
750	Loi	Good	GSM150977.CEL.gz	68.97	0	-	-
751	Loi	Good	GSM150978.CEL.gz	60.97	0	-	-
752	Loi	Good	GSM150979.CEL.gz	66.48	0	-	-
753	Loi	-	GSM150980.CEL.gz	53.84	0	-	-
754	Loi	-	GSM150981.CEL.gz	53.35	0	-	-
755	Loi	-	GSM150982.CEL.gz	53.58	-	-	-
756	Loi	Good	GSM150983.CEL.gz	123.52	0	-	-
757	Loi	Good	GSM150984.CEL.gz	110.06	0	-	-
758	Loi	Good	GSM150985.CEL.gz	124.58	0	-	-
759	Loi	Good	GSM150986.CEL.gz	119.61	0	-	-
760	Loi	Poor	GSM150987.CEL.gz	44.42	1	-	-
761	Loi	-	GSM150988.CEL.gz	0.26	-	-	-
762	Loi	Good	GSM150989.CEL.gz	61.13	0	-	-
763	Loi	Good	GSM150990.CEL.gz	115.42	0	-	-
764	Loi	Good	GSM150991.CEL.gz	150.06	0	-	-
765	Loi	Good	GSM150992.CEL.gz	115.19	0	-	-
766	Loi	Good	GSM150993.CEL.gz	112.77	0	-	-
767	Loi	Good	GSM150994.CEL.gz	130.13	0	-	-
768	Loi	Good	GSM150995.CEL.gz	122.26	0	-	-
769	Loi	Good	GSM150996.CEL.gz	117.81	0	-	-
770	Loi	Good	GSM150997.CEL.gz	116.29	0	-	-
771	Loi	Poor	GSM150998.CEL.gz	31.45	1	-	-
772	Loi	Poor	GSM150999.CEL.gz	41.71	1	-	-
773	Loi	-	GSM151000.CEL.gz	51.16	0	-	-
774	Loi	Poor	GSM151001.CEL.gz	15.9	1	-	-
775	Loi	Good	GSM151002.CEL.gz	71.13	0	-	-
776	Loi	Good	GSM151003.CEL.gz	64.32	0	-	-
777	Loi	-	GSM151004.CEL.gz	48.35	0	-	-
778	Loi	Good	GSM151005.CEL.gz	111.06	0	-	-
779	Loi	-	GSM151006.CEL.gz	30.55	0	-	-
780	Loi	-	GSM151007.CEL.gz	48.9	0	-	-
781	Loi	Good	GSM151008.CEL.gz	97.06	0	-	-
782	Loi	Good	GSM151009.CEL.gz	95.26	0	-	-
783	Loi	Good	GSM151010.CEL.gz	94.19	0	-	-
784	Loi	Good	GSM151011.CEL.gz	95.52	0	-	-
785	Loi	-	gsm65340.cel.gz	27.42	0	-	-
786	Loi	-	gsm65341.cel.gz	34.97	0	-	-
787	Loi	-	gsm65342.cel.gz	2.13	-	-	-
788	Loi	-	gsm65343.cel.gz	7.29	-	-	-
789	Loi	-	gsm65344.cel.gz	8.35	0	-	-
790	Loi	-	gsm65345.cel.gz	15.9	0	-	-
791	Loi	-	gsm65346.cel.gz	18.29	0	-	-
792	Loi	-	gsm65347.cel.gz	29.84	-	-	-
793	Loi	-	gsm65348.cel.gz	30.61	0	-	-
794	Loi	-	gsm65349.cel.gz	32.29	-	-	-
795	Loi	-	gsm65350.cel.gz	43.97	-	-	-
796	Loi	-	gsm65351.cel.gz	45.68	0	-	-
797	Loi	-	gsm65352.cel.gz	49.13	0	-	-
798	Loi	-	gsm65353.cel.gz	52.9	0	-	-
799	Loi	-	gsm65354.cel.gz	58.32	0	-	-
800	Loi	Good	gsm65355.cel.gz	60.81	0	-	-
801	Loi	Good	gsm65356.cel.gz	62.61	0	-	-
802	Loi	Good	gsm65357.cel.gz	64.55	0	-	-
803	Loi	Good	gsm65358.cel.gz	65.71	0	-	-
804	Loi	Good	gsm65359.cel.gz	67.55	0	-	-
805	Loi	Good	gsm65360.cel.gz	82.48	0	-	-
806	Loi	Good	gsm65361.cel.gz	86.61	0	-	-
807	Loi	Good	gsm65362.cel.gz	89.23	0	-	-
808	Loi	Good	gsm65363.cel.gz	101.16	0	-	-
809	Loi	Good	gsm65364.cel.gz	103.71	0	-	-
810	Loi	Good	gsm65365.cel.gz	104.58	0	-	-
811	Loi	Good	gsm65366.cel.gz	110.84	0	-	-
812	Loi	Good	gsm65367.cel.gz	116.32	0	-	-
813	Loi	Good	gsm65368.cel.gz	121.13	0	-	-

814	Loi	-	gsm65369.cel.gz	5.87	-	-	-
815	Loi	Poor	gsm65370.cel.gz	20.48	1	-	-
816	Loi	Poor	gsm65371.cel.gz	18.19	1	-	-
817	Loi	Poor	gsm65372.cel.gz	30.26	1	-	-
818	Loi	-	gsm65373.cel.gz	76.55	1	-	-
819	Loi	Poor	gsm65374.cel.gz	53.32	1	-	-
820	Loi	-	gsm65375.cel.gz	57.55	-	-	-
821	Loi	-	gsm65376.cel.gz	58.87	0	-	-
822	Loi	-	gsm65377.cel.gz	66.77	1	-	-
823	Loi	-	gsm65378.cel.gz	83.87	1	-	-
824	Loi	Good	gsm65379.cel.gz	95.42	0	-	-
825	Chin	-	E-TABM-158-RAW-CEL-1639356117.CEL	25.2	0	-	-
826	Chin	Good	E-TABM-158-RAW-CEL-1639356804.CEL	135.84	0	-	-
827	Chin	-	E-TABM-158-RAW-CEL-1639358483.CEL	18.48	0	-	-
828	Chin	-	E-TABM-158-RAW-CEL-1639357292.CEL	4.92	0	-	-
829	Chin	-	E-TABM-158-RAW-CEL-1639358420.CEL	49.92	0	-	-
830	Chin	-	E-TABM-158-RAW-CEL-1639356160.CEL	36.72	0	-	-
831	Chin	-	E-TABM-158-RAW-CEL-1639358312.CEL	22.08	0	-	-
832	Chin	Poor	E-TABM-158-RAW-CEL-1639356292.CEL	4.08	1	-	-
833	Chin	-	E-TABM-158-RAW-CEL-1639356484.CEL	12.6	0	-	-
834	Chin	-	E-TABM-158-RAW-CEL-1639357532.CEL	56.52	0	-	-
835	Chin	Good	E-TABM-158-RAW-CEL-1639357692.CEL	67.32	0	-	-
836	Chin	-	E-TABM-158-RAW-CEL-1639357932.CEL	-	-	-	-
837	Chin	-	E-TABM-158-RAW-CEL-1639356009.CEL	52.68	0	-	-
838	Chin	-	E-TABM-158-RAW-CEL-1639357646.CEL	29.52	0	-	-
839	Chin	-	E-TABM-158-RAW-CEL-1639358084.CEL	8.04	0	-	-
840	Chin	-	E-TABM-158-RAW-CEL-1639358188.CEL	9.84	0	-	-
841	Chin	-	E-TABM-158-RAW-CEL-1639357800.CEL	1.56	0	-	-
842	Chin	-	E-TABM-158-RAW-CEL-1639358104.CEL	32.88	0	-	-
843	Chin	-	E-TABM-158-RAW-CEL-1639356137.CEL	16.32	0	-	-
844	Chin	-	E-TABM-158-RAW-CEL-1639358332.CEL	13.56	0	-	-
845	Chin	Good	E-TABM-158-RAW-CEL-1639356930.CEL	90.72	0	-	-
846	Chin	-	E-TABM-158-RAW-CEL-1639355881.CEL	24.6	0	-	-
847	Chin	-	E-TABM-158-RAW-CEL-1639358376.CEL	18.12	0	-	-
848	Chin	Good	E-TABM-158-RAW-CEL-1639357226.CEL	136.2	0	-	-
849	Chin	-	E-TABM-158-RAW-CEL-1639356336.CEL	5.16	0	-	-
850	Chin	Poor	E-TABM-158-RAW-CEL-1639357976.CEL	3.72	1	-	-
851	Chin	Good	E-TABM-158-RAW-CEL-1639356033.CEL	72.24	0	-	-
852	Chin	Poor	E-TABM-158-RAW-CEL-1639356992.CEL	5.04	1	-	-
853	Chin	Good	E-TABM-158-RAW-CEL-1639356073.CEL	104.04	0	-	-
854	Chin	Good	E-TABM-158-RAW-CEL-1639357076.CEL	67.68	0	-	-
855	Chin	Good	E-TABM-158-RAW-CEL-1639358268.CEL	131.76	0	-	-
856	Chin	Good	E-TABM-158-RAW-CEL-1639357400.CEL	81.12	0	-	-
857	Chin	-	E-TABM-158-RAW-CEL-1639356632.CEL	58.08	0	-	-
858	Chin	Good	E-TABM-158-RAW-CEL-1639357996.CEL	128.64	0	-	-
859	Chin	Poor	E-TABM-158-RAW-CEL-1639356758.CEL	8.04	1	-	-
860	Chin	Good	E-TABM-158-RAW-CEL-1639355965.CEL	119.76	0	-	-
861	Chin	Poor	E-TABM-158-RAW-CEL-1639358352.CEL	51.96	1	-	-
862	Chin	Poor	E-TABM-158-RAW-CEL-1639358228.CEL	22.32	1	-	-
863	Chin	-	E-TABM-158-RAW-CEL-1639357098.CEL	29.76	0	-	-
864	Chin	-	E-TABM-158-RAW-CEL-1639356846.CEL	34.8	0	-	-
865	Chin	Good	E-TABM-158-RAW-CEL-1639356204.CEL	94.32	0	-	-
866	Chin	Good	E-TABM-158-RAW-CEL-1639356420.CEL	63.36	0	-	-
867	Chin	Good	E-TABM-158-RAW-CEL-1639357380.CEL	67.56	0	-	-
868	Chin	-	E-TABM-158-RAW-CEL-1639357356.CEL	78.24	1	-	-
869	Chin	Good	E-TABM-158-RAW-CEL-1639357184.CEL	124.32	0	-	-
870	Chin	Good	E-TABM-158-RAW-CEL-1639358016.CEL	89.64	0	-	-
871	Chin	Poor	E-TABM-158-RAW-CEL-1639355793.CEL	0	1	-	-
872	Chin	Good	E-TABM-158-RAW-CEL-1639358060.CEL	135.36	0	-	-
873	Chin	Good	E-TABM-158-RAW-CEL-1639356228.CEL	71.76	0	-	-
874	Chin	Good	E-TABM-158-RAW-CEL-1639356588.CEL	91.92	0	-	-
875	Chin	-	E-TABM-158-RAW-CEL-1639357712.CEL	81.48	1	-	-
876	Chin	Good	E-TABM-158-RAW-CEL-1639357248.CEL	125.4	0	-	-
877	Chin	Good	E-TABM-158-RAW-CEL-1639355813.CEL	96.12	0	-	-
878	Chin	Good	E-TABM-158-RAW-CEL-1639357034.CEL	131.76	0	-	-
879	Chin	Good	E-TABM-158-RAW-CEL-1639356612.CEL	69.48	0	-	-
880	Chin	Good	E-TABM-158-RAW-CEL-1639356886.CEL	115.2	0	-	-
881	Chin	Good	E-TABM-158-RAW-CEL-1639357848.CEL	109.68	0	-	-
882	Chin	Good	E-TABM-158-RAW-CEL-1639355709.CEL	72.24	0	-	-
883	Chin	Good	E-TABM-158-RAW-CEL-1639356272.CEL	141.36	0	-	-
884	Chin	Good	E-TABM-158-RAW-CEL-1639357556.CEL	94.8	0	-	-
885	Chin	Good	E-TABM-158-RAW-CEL-1639356097.CEL	123.6	0	-	-
886	Chin	Good	E-TABM-158-RAW-CEL-1639358208.CEL	122.4	0	-	-
887	Chin	Good	E-TABM-158-RAW-CEL-1639356441.CEL	98.16	0	-	-

888	Chin	Good	E-TABM-158-RAW-CEL-1639357014.CEL	82.08	0	-	-
889	Chin	-	E-TABM-158-RAW-CEL-1639357600.CEL	53.64	0	-	-
890	Chin	-	E-TABM-158-RAW-CEL-1639357512.CEL	25.56	0	-	-
891	Chin	Good	E-TABM-158-RAW-CEL-1639357668.CEL	102.12	0	-	-
892	Chin	Poor	E-TABM-158-RAW-CEL-1639356824.CEL	48.48	1	-	-
893	Chin	-	E-TABM-158-RAW-CEL-1639357315.CEL	4.2	0	-	-
894	Chin	Good	E-TABM-158-RAW-CEL-1639357140.CEL	116.52	0	-	-
895	Chin	-	E-TABM-158-RAW-CEL-1639357204.CEL	59.64	0	-	-
896	Chin	Poor	E-TABM-158-RAW-CEL-1639356312.CEL	29.64	1	-	-
897	Chin	Good	E-TABM-158-RAW-CEL-1639357624.CEL	100.56	0	-	-
898	Chin	Poor	E-TABM-158-RAW-CEL-1639356184.CEL	34.56	1	-	-
899	Chin	Good	E-TABM-158-RAW-CEL-1639357580.CEL	143.52	0	-	-
900	Chin	Good	E-TABM-158-RAW-CEL-1639356676.CEL	111	0	-	-
901	Chin	Poor	E-TABM-158-RAW-CEL-1639356548.CEL	13.8	1	-	-
902	Chin	-	E-TABM-158-RAW-CEL-1639357912.CEL	36.84	0	-	-
903	Chin	Poor	E-TABM-158-RAW-CEL-1639356248.CEL	0	1	-	-
904	Chin	Good	E-TABM-158-RAW-CEL-1639357956.CEL	108.12	0	-	-
905	Chin	Good	E-TABM-158-RAW-CEL-1639357056.CEL	96.96	0	-	-
906	Chin	Good	E-TABM-158-RAW-CEL-1639357756.CEL	76.32	0	-	-
907	Chin	Good	E-TABM-158-RAW-CEL-1639356524.CEL	66.84	0	-	-
908	Chin	Good	E-TABM-158-RAW-CEL-1639356464.CEL	113.64	0	-	-
909	Chin	Poor	E-TABM-158-RAW-CEL-1639356908.CEL	0	1	-	-
910	Chin	Good	E-TABM-158-RAW-CEL-1639356356.CEL	154.44	0	-	-
911	Chin	Good	E-TABM-158-RAW-CEL-1639357118.CEL	136.2	0	-	-
912	Chin	-	E-TABM-158-RAW-CEL-1639355729.CEL	52.68	0	-	-
913	Chin	Poor	E-TABM-158-RAW-CEL-1639356780.CEL	32.52	1	-	-
914	Chin	Good	E-TABM-158-RAW-CEL-1639356053.CEL	121.08	0	-	-
915	Chin	Good	E-TABM-158-RAW-CEL-1639357336.CEL	107.52	0	-	-
916	Chin	Good	E-TABM-158-RAW-CEL-1639356568.CEL	80.76	0	-	-
917	Chin	Poor	E-TABM-158-RAW-CEL-1639357444.CEL	8.04	1	-	-
918	Chin	Good	E-TABM-158-RAW-CEL-1639358144.CEL	106.92	0	-	-
919	Chin	-	E-TABM-158-RAW-CEL-1639357736.CEL	25.32	0	-	-
920	Chin	Good	E-TABM-158-RAW-CEL-1639358440.CEL	165	0	-	-
921	Chin	-	E-TABM-158-RAW-CEL-1639356952.CEL	39.84	0	-	-
922	Chin	Poor	E-TABM-158-RAW-CEL-1639355773.CEL	15.84	1	-	-
923	Chin	-	E-TABM-158-RAW-CEL-1639356380.CEL	18.84	0	-	-
924	Chin	Good	E-TABM-158-RAW-CEL-1639355943.CEL	115.2	0	-	-
925	Chin	Good	E-TABM-158-RAW-CEL-1639357780.CEL	124.08	0	-	-
926	Chin	Good	E-TABM-158-RAW-CEL-1639358460.CEL	68.4	0	-	-
927	Chin	Good	E-TABM-158-RAW-CEL-1639356696.CEL	153.36	0	-	-
928	Chin	Good	E-TABM-158-RAW-CEL-1639355985.CEL	123.72	0	-	-
929	Chin	Good	E-TABM-158-RAW-CEL-1639358396.CEL	134.88	0	-	-
930	Chin	Poor	E-TABM-158-RAW-CEL-1639357162.CEL	42.12	1	-	-
931	Chin	Good	E-TABM-158-RAW-CEL-1639355921.CEL	117.6	0	-	-
932	Chin	-	E-TABM-158-RAW-CEL-1639356972.CEL	10.32	0	-	-
933	Chin	Good	E-TABM-158-RAW-CEL-1639357424.CEL	133.2	0	-	-
934	Chin	Poor	E-TABM-158-RAW-CEL-1639357468.CEL	27.24	1	-	-
935	Chin	-	E-TABM-158-RAW-CEL-1639355901.CEL	17.04	0	-	-
936	Chin	Poor	E-TABM-158-RAW-CEL-1639356736.CEL	0	1	-	-
937	Chin	Good	E-TABM-158-RAW-CEL-1639356504.CEL	109.2	0	-	-
938	Chin	Poor	E-TABM-158-RAW-CEL-1639355859.CEL	43.44	1	-	-
939	Chin	Good	E-TABM-158-RAW-CEL-1639358124.CEL	123.12	0	-	-
940	Chin	Poor	E-TABM-158-RAW-CEL-1639357488.CEL	0	1	-	-
941	Chin	Poor	E-TABM-158-RAW-CEL-1639355750.CEL	18.36	1	-	-
942	Chin	Good	E-TABM-158-RAW-CEL-1639355837.CEL	73.44	0	-	-
943	Chin	Good	E-TABM-158-RAW-CEL-1639356716.CEL	102.36	0	-	-
944	Chin	Good	E-TABM-158-RAW-CEL-1639356652.CEL	83.28	0	-	-
945	Chin	Poor	E-TABM-158-RAW-CEL-1639358292.CEL	25.8	1	-	-
946	Chin	Good	E-TABM-158-RAW-CEL-1639358040.CEL	119.28	0	-	-
947	Chin	-	E-TABM-158-RAW-CEL-1639356866.CEL	36.12	0	-	-
