# Supplementary material for: A comprehensive sensitivity analysis of microarray breast cancer classification under feature variability
Source: BMC Bioinformatics. 2009 Nov 26;10:389. doi: 10.1186/1471-2105-10-389 (PMC2789744; doi:10.1186/1471-2105-10-389)
Supplement: Additional file 2 — Overview of additional 87 hybridizations from the Van de Vijver. The column SampleID indicates the identifiers as used by Van de Vijver et al. [34]. The selected hybridizations represent all lymph-node negative cases that were not yet contained in the original publication by Van 't Veer et al. [2]. [file 1471-2105-10-389-S2.PDF]

Count	SampleID	EVENTmeta	TIMEmeta	ClassLabel
1	103	1	4.95	Poor
2	107	1	2.54	Poor
3	109	1	3.2	Poor
4	110	1	2.17	Poor
5	111	1	1.27	Poor
6	113	1	1	Poor
7	118	0	-	Good
8	120	0	-	Good
9	126	1	6.32	Good
10	131	0	-	Good
11	140	0	-	Good
12	142	0	-	Good
13	144	0	-	Good
14	148	0	-	Good
15	153	1	1.18	Poor
16	154	0	-	Good
17	155	1	0.93	Poor
18	164	0	-	Good
19	165	1	10.44	Good
20	167	0	-	Good
21	175	1	7.59	Good
22	179	0	-	Good
23	181	0	-	Good
24	183	0	-	Good
25	185	0	-	Good
26	189	0	-	Good
27	191	0	-	Good
28	193	0	-	Good
29	199	0	-	Good
30	201	0	-	Good
31	202	1	3.38	Poor
32	205	0	-	Good
33	207	0	-	Good
34	212	0	-	Good
35	214	1	7.48	Good
36	215	0	-	Good
37	219	0	-	Good
38	226	0	-	Good
39	233	0	-	Good
40	235	0	-	Good
41	239	0	-	Good
42	246	0	-	Good
43	248	0	-	Good
44	259	1	5.52	Good
45	266	0	-	Good
46	268	0	-	Good
47	270	0	-	Good
48	271	0	-	Good
49	274	0	-	Good

50	278	0	-	Good
51	285	0	-	Good
52	286	0	-	Good
53	295	0	-	Good
54	296	0	-	Good
55	302	0	-	Good
56	304	1	6.71	Good
57	305	0	-	Good
58	306	0	-	Good
59	312	0	-	Good
60	313	1	6.06	Good
61	319	1	6.37	Good
62	323	0	-	Good
63	326	0	-	Good
64	329	0	-	Good
65	331	1	2.16	Poor
66	333	0	-	Good
67	336	0	-	Good
68	338	0	-	Good
69	339	0	-	Good
70	344	0	-	Good
71	348	0	-	Good
72	349	0	-	Good
73	352	0	-	Good
74	354	0	-	Good
75	355	0	-	Good
76	356	0	-	Good
77	358	0	-	Good
78	364	0	-	Good
79	365	0	-	Good
80	366	0	-	Good
81	367	1	0.57	Poor
82	368	1	9.57	Good
83	369	1	3.26	Poor
84	388	0	-	Good
85	391	0	-	Good
86	394	0	-	Good
87	402	0	-	Good
