# Supplementary material for: A comprehensive sensitivity analysis of microarray breast cancer classification under feature variability
Source: BMC Bioinformatics. 2009 Nov 26;10:389. doi: 10.1186/1471-2105-10-389 (PMC2789744; doi:10.1186/1471-2105-10-389)
Supplement: Additional file 3 — Supplementary information on perturbation schemes and classifiers. The file contains additional information on how to construct perturbed expression profiles for MAS5.0, dChip and the Rosetta data. In addition, information on parameter settings for the SVM and RF classifiers is provided. [file 1471-2105-10-389-S3.PDF]

# Additional File 3: Supplementary information on perturbation schemes and classifiers

Herman MJ Sontrop*[1], Perry D Moerland[2], René van den Ham[3], Marcel JT Reinders[4], Wim FJ Verhaegh[1]

[1]Molecular Diagnostics Department, Philips Research, High Tech Campus 12a, 5656 AE Eindhoven, The Netherlands
[2]Bioinformatics Laboratory, Department of Clinical Epidemiology, Biostatistics and Bioinformatics, Academic Medical Center, Meibergdreef 9, 1100 AZ Amsterdam, The Netherlands
[3]Biomolecular Engineering Department, Philips Research, High Tech Campus 11, 5656 AE Eindhoven, The Netherlands
[4]Delft Bioinformatics Lab, Delft University of Technology, Mekelweg 4, 2628 CD Delft, The Netherlands

Email: Herman MJ Sontrop*- herman.sontrop@philips.com; Perry D Moerland - p.d.moerland@amc.uva.nl; René van den Ham - rene.van.den.ham@philips.com; Marcel JT Reinders - m.j.t.reinders@tudelft.nl; Wim FJ Verhaegh - wim.verhaegh@philips.com;

*Corresponding author

## MAS5.0: preprocessing and perturbation

In order to obtain perturbed expression values, we use the standard error of the probeset expression summary of MAS 5.0. The summarization step in MAS 5.0 works as follows [1, 2]. Let the natural scale intensities of the different probes in a certain probeset and on a certain array be given by $\{y_1, y_2, \ldots, y_n\}$, with $n$ denoting the number of probes in the probeset. Assume that these intensities are already background- and PM-corrected via bg.correct and pmcorrect.mas in the affy package. In order to create a summary value for a probeset, MAS 5.0 calculates a robust average using the 1-step Tukey biweight estimator on $\log_2$ scale. The 1-step Tukey biweight estimator calculates a weighted average of the individual probes, using the bisquare function as a means to generate weights

$$w_k = \begin{cases} 0 & \text{if } |u_k| > 1 \\ (1 - u_k^2)^2 & \text{if } |u_k| \leq 1 \end{cases} \tag{1}$$

where $u_k$ is defined as

$$u_k = \frac{\log_2(y_k) - M}{cS + \epsilon}. \tag{2}$$

with $M$ the median of the $\log_2(y_k)$ values and $S$ the median of the absolute deviations from $M$. Furthermore, $c$ and $\epsilon$ are tuning constants, with default values of 5 and 0.0001, respectively. The probeset

summary $\tau$ is obtained by creating the weighted average

$$\tau = \frac{\sum_{k=1}^{n} w_k \log_2(y_k)}{\sum_{k=1}^{n} w_k}. \tag{3}$$

using function tukey.biweight in expresso.

The MAS5.0 preprocessing cascade ends with an intensity independent normalization step, in which each probeset summary on array $i$ is multiplied by a scaling factor $\theta_i$, such that the average intensity over all probeset summaries (on the natural scale) reaches a certain target intensity. The expression summary returned by MAS5.0 for probeset $j$ on array $i$ then equals

$$2^{\tau_{ij}} \cdot \theta_i. \tag{4}$$

Similarly to one of the original studies in the compendium [3], we scaled all arrays to a target intensity of 600 using the function affy.scalevalue.exprSet.

We can compute the parametric based standard error $\sigma_\tau$ of $\tau$ as described in [1, 2]

$$\sigma_\tau = \frac{\sqrt{\sum_{k=1,|u_k|\leq 1}^{n} (\log_2(y_k) - x)^2 (1 - u_k^2)^4}}{|\sum_{k=1,|u_k|\leq 1}^{n} (1 - u_k^2)(1 - 5u_k^2)|}. \tag{5}$$

In [1, 2] it is suggested that perturbed $\tau$ values, denoted by $\widetilde{\tau}$, can be obtained by

$$\widetilde{\tau} = \tau + rt(\nu) \cdot \sigma_\tau, \tag{6}$$

where $rt(\nu)$ represents a random draw from a t-distribution with $\nu$ degrees of freedom. The value for $\nu$ is set equal to $\max\{0.7(n-1), 1\}$; see [1]. Hence after $\log_2$ transformation and median centering, perturbed MAS5.0 expression values $\widetilde{x}_{ij}$, for array $i$ and probeset $j$, can be computed using Equations (4) and (6) as

$$\widetilde{x}_{ij} = \tau_{ij} + rt(\nu_{ij}) \cdot \sigma_{\tau_{ij}} + \log_2(\theta_i) - \text{med}_j. \tag{7}$$

where $med_j$ represents the median expression level of gene $j$ taken over all available hybridizations (see column *total* in Table 1 of the main text).

## dChip: preprocessing and perturbation

We used the PM-only version of dChip [4]. In this case, all estimated expression values are strictly positive and we can log-transform the data. Similarly to the default choice in dChip, we maintained a floor of one on the intensity data before log transformation. In rare cases where negative values arise after perturbing dChip-preprocessed data (last three columns of Table 1), we repaired the intensity value by

| *author* | *all* | *all*$_1$ | *all*$_2$ | *lab* | *lab*$_1$ | *lab*$_2$ | *fsel* | *fsel*$_1$ | *fsel*$_2$ |
|---|---|---|---|---|---|---|---|---|---|
| Desmedt | 0 | 0.15 | 0.97 | 0 | 0.16 | 1.02 | 0 | 0.08 | 1.17 |
| Minn | 0 | 0.11 | 0.88 | 0 | 0.11 | 0.87 | 0 | 0.16 | 1.55 |
| Miller | 0 | 0.07 | 0.45 | 0 | 0.07 | 0.45 | 0 | 0.04 | 0.48 |
| Pawitan | 0 | 0.28 | 0.99 | 0 | 0.29 | 0.97 | 0 | 0.12 | 0.72 |
| Loi | 0 | 0.03 | 0.40 | 0 | 0.04 | 0.43 | 0 | 0.24 | 1.82 |
| Chin | 0 | 0.15 | 1.26 | 0 | 0.12 | 1.17 | 0 | 0.09 | 1.63 |

Table 1: Overview negative values in dChip. For each of the six datasets the column *all* gives the percentage of negative values, when taken over all probeset summaries and all available hybridizations. The columns *all*$_1$, and *all*$_2$ provide the same information as in *all*, on data for which we subtracted for each individual entry, one or two times the corresponding estimated standard error, respectively. The columns *lab*, *lab*$_1$, and *lab*$_2$ provide the same information as the previous three columns, when using only hybridizations that have a properly defined class label. Finally, columns *fsel*, *fsel*$_1$, and *fsel*$_2$ provide similar statistics, when using only the top-100 ranked features for each split, as obtained from the multi-rank strategy as described in the main text. The numbers represent averages over 50 splits.

setting it equal to one. Hence log-transformed median centered perturbed expression values $\widetilde{x}_{ij}$ for dChip expression estimates were computed as

$$\widetilde{x}_{ij} = \log_2(\max\{x_{ij} + \epsilon_{ij}, 1\}) - \text{med}_j \qquad \text{with} \qquad \epsilon_{ij} \sim N(0, \sigma_{ij}). \tag{8}$$

## Rosetta data: perturbation

For the Rosetta data, the technical noise levels were estimated by applying the Rosetta error model [5]. This yields, for each sample $i$ and each gene $j$, a log-ratio expression measurement that is normally distributed with mean $x_{ij}$ and standard deviation $\sigma_{ij}$. For the data of Van de Vijver $\sigma_{ij}$ was reported directly. However, for the data of Van 't Veer only $x_{ij}$ and the $p$-value $p_{ij}$ of observing a value for the log ratio more extreme than $x_{ij}$ if the true log ratio is zero with measurement error $\sigma_{ij}$ were given. In this case, we can compute $\sigma_{ij}$ as

$$\sigma_{ij} = \frac{|x_{ij}|}{\text{cdf}^{-1}(1 - \frac{p_{ij}}{2})}, \tag{9}$$

where cdf denotes the cumulative density function of a $N(0,1)$ distributed random variable. Equation (9) can be obtained by noting that for a normally distributed stochast $X$ with mean 0 and standard deviation $\sigma$ the two-sided $p$-value is given by

$$p = 2 \cdot \Pr(X > |x|) = 2 \cdot (1 - \Pr(X \leq |x|)) = 2 \cdot (1 - \Pr(\frac{X}{\sigma} \leq \frac{|x|}{\sigma})) = 2 \cdot (1 - \text{cdf}(\frac{|x|}{\sigma})). \tag{10}$$

Although Equation (9) provides the value of $\sigma_{ij}$ for most pairs of $x_{ij}$ and $p_{ij}$, in two cases we cannot deduce the value of $\sigma_{ij}$ using (9). When $p_{ij}$ equals 1 the denominator of (9) equals zero and hence the

outcome is undefined. In that case, we conservatively set $\sigma_{ij}$ to zero. Furthermore, we have to be careful of rounding effects if $p_{ij}$ is close to one, since then the denominator of (9) is close to zero, which may yield unrealistically high values for $\sigma_{ij}$. To avoid this, we bound $\sigma_{ij}$ from above by one. Since all reported expression values involved in the dataset of Van 't Veer are between $-2$ and 2, it makes sense to generate expression values in a similar range. A value of $\sigma_{\max} = 1$ ensures that with high probability we will generate a perturbed expression value between $-2$ and 2 for a gene with $x_{ij}$ equal to zero. The same threshold was applied to the estimates corresponding to the study by Van de Vijver. Finally, for the Rosetta data, given the resulting standard deviations $\sigma_{ij}$, we perturbed the gene expression data by simply adding to each measurement $x_{ij}$ some Gaussian noise with mean zero and standard deviation $\sigma_{ij}$:

$$\widetilde{x}_{ij} = x_{ij} + \epsilon_{ij} \qquad \text{with} \qquad \epsilon_{ij} \sim N(\mu = 0, \sigma = \min\{\sigma_{\max}, \sigma_{ij}\}). \tag{11}$$

## Classifiers

### Nearest mean classification using cosine distance

Let $I^{\mathrm{g}}$ denote the set of $n_{\mathrm{g}}$ samples belonging to the good prognosis class and $I^{\mathrm{p}}$ denote the set of $n_{\mathrm{p}}$ samples belonging to the poor prognosis class. The average good profile $\mathbf{m}^{\mathrm{g}}$ and the average poor profile $\mathbf{m}^{\mathrm{p}}$ are defined as

$$m_j^{\mathrm{g}} = \frac{1}{n_{\mathrm{g}}} \sum_{i \in I^{\mathrm{g}}} x_{ij} \qquad \text{and} \qquad m_j^{\mathrm{p}} = \frac{1}{n_{\mathrm{p}}} \sum_{i \in I^{\mathrm{p}}} x_{ij}.$$

A nearest mean classifier, using cosine correlation as its distance measure classifies a sample $\mathbf{x}$ to the good prognosis class if the distance of $\mathbf{x}$ to $\mathbf{m}^{\mathrm{g}}$, denoted by $d(\mathbf{x}, \mathbf{m}^{\mathrm{g}})$, is smaller than the distance of $\mathbf{x}$ to $\mathbf{m}^{\mathrm{p}}$, denoted by $d(\mathbf{x}, \mathbf{m}^{\mathrm{p}})$. From the definition of cosine distance it follows that a sample will be classified as having a good prognosis if and only if

$$\begin{aligned} d(\mathbf{x}, \mathbf{m}^{\mathrm{g}}) &< d(\mathbf{x}, \mathbf{m}^{\mathrm{p}}) \\ 1 - \frac{\mathbf{x}^T \mathbf{m}^{\mathrm{g}}}{\|\mathbf{x}\| \|\mathbf{m}^{\mathrm{g}}\|} &< 1 - \frac{\mathbf{x}^T \mathbf{m}^{\mathrm{p}}}{\|\mathbf{x}\| \|\mathbf{m}^{\mathrm{p}}\|} \\ \mathbf{x}^T \left(\frac{\mathbf{m}^{\mathrm{g}}}{\|\mathbf{m}^{\mathrm{g}}\|} - \frac{\mathbf{m}^{\mathrm{p}}}{\|\mathbf{m}^{\mathrm{p}}\|}\right) &> 0 \\ \mathbf{x}^T \mathbf{w} &> 0, \end{aligned} \tag{12}$$

where we define

$$\mathbf{w} = \frac{\mathbf{m}^{\mathrm{g}}}{\|\mathbf{m}^{\mathrm{g}}\|} - \frac{\mathbf{m}^{\mathrm{p}}}{\|\mathbf{m}^{\mathrm{p}}\|}.$$

Note that the classification rule (12) results in a linear classifier. The left-hand side of Equation (12) is usually referred to as the *discriminant score*.

## Parameter settings

*Support vector machines*

SVM results were obtained using the R package e1071. For each feature set, a grid search was performed to find the best parameters using the function tune. The parameter cost was varied from 0.5 to 5, with increments of 0.5. In addition, when using a radial basis kernel function, the parameter gamma equaled $\frac{2^x}{k}$, with $x$ varying from $-3$ to 3, with increments of 1. Here $k$ represents the number of features, which in our experiments varied between 1 and 100. Best parameters were selected using 3-fold cross validation, using tune.control. The parameter class.weights was used to set weights inversely proportional to the class frequencies, in order to compensate for the unbalanced class distributions.

*Random forests*

Random forest results were obtained using the R package randomForest. In randomForest the parameter ntree was set to 1000, while the parameters mtree and nodesize were set to their default values, the square root of the number of features and 1, respectively. During training the parameter sampsize was used in combination with the parameter strata to ward against unbalanced class distributions, by drawing an equal number of samples from the good and poor prognosis cases. In our case the number of samples to be drawn was always equal to the number of poor prognosis cases in the training set.

## References


1. Affymetrix: *Statistical Algorithms Reference Guide* 2002, [http://www.affymetrix.com/support/technical/whitepapers/sadd_whitepaper.pdf].
2. Bolstad B: **Low-level analysis of high-density oligonucleotide array data: background, normalization and summarization**. *PhD thesis*, University of California 2004.
3. Desmedt C, Piette F, Loi S, Wang Y, Lallemand F, Haibe-Kains B, Viale G, Delorenzi M, Zhang Y, d'Assignies M, et al.: **Strong time dependence of the 76-Gene prognostic signature for node-negative breast cancer patients in the TRANSBIG multicenter independent validation series**. *Clinical Cancer Research* 2007, **13**(11):3207–3214.
4. Li C, Wong W: **Model-based analysis of oligonucleotide arrays: expression index computation and outlier detection**. *Proceedings of the National Academy of Sciences* 2001, **98**:31–36.
5. Weng L, Dai H, Zhan Y, He Y, Stepaniants S, Bassett D: **Rosetta error model for gene expression analysis**. *Bioinformatics* 2006, **22**(9):1111–1121.
